# Formation of left-handed helices by C2′-fluorinated nucleic acids under physiological salt conditions

**DOI:** 10.1093/nar/gkae508

**Published:** 2024-06-14

**Authors:** Roberto El-Khoury, Cristina Cabrero, Santiago Movilla, Harneesh Kaur, David Friedland, Arnau Domínguez, James D Thorpe, Morgane Roman, Modesto Orozco, Carlos González, Masad J Damha

**Affiliations:** Department of Chemistry, McGill University, Montreal, Quebec H3A 0B8, Canada; Instituto de Química Física Blas Cabrera, CSIC, Serrano 119, 28006 Madrid, Spain; Institute for Research in Biomedicine (IRB Barcelona), The Barcelona Institute of Science and Technology (BIST), 08028 Barcelona, Spain; Department of Chemistry, McGill University, Montreal, Quebec H3A 0B8, Canada; Department of Chemistry, McGill University, Montreal, Quebec H3A 0B8, Canada; Department of Chemistry, McGill University, Montreal, Quebec H3A 0B8, Canada; IQAC-CSIC, Jordi Girona 18-26, 08034 Barcelona, Spain; Department of Chemistry, McGill University, Montreal, Quebec H3A 0B8, Canada; Department of Chemistry, McGill University, Montreal, Quebec H3A 0B8, Canada; Institute for Research in Biomedicine (IRB Barcelona), The Barcelona Institute of Science and Technology (BIST), 08028 Barcelona, Spain; Instituto de Química Física Blas Cabrera, CSIC, Serrano 119, 28006 Madrid, Spain; Department of Chemistry, McGill University, Montreal, Quebec H3A 0B8, Canada

## Abstract

Recent findings in cell biology have rekindled interest in Z-DNA, the left-handed helical form of DNA. We report here that two minimally modified nucleosides, 2′F-araC and 2′F-riboG, induce the formation of the Z-form under low ionic strength. We show that oligomers entirely made of these two nucleosides exclusively produce left-handed duplexes that bind to the Zα domain of ADAR1. The effect of the two nucleotides is so dramatic that Z-form duplexes are the only species observed in 10 mM sodium phosphate buffer and neutral pH, and no B-form is observed at any temperature. Hence, in contrast to other studies reporting formation of Z/B-form equilibria by a preference for purine glycosidic angles in *syn*, our NMR and computational work revealed that sequential 2′F**^…^**H_2_N and intramolecular 3′H**^…^**N3′ interactions stabilize the left-handed helix. The equilibrium between B- and Z- forms is slow in the ^19^F NMR time scale (≥ms), and each conformation exhibited unprecedented chemical shift differences in the ^19^F signals. This observation led to a reliable estimation of the relative population of B and Z species and enabled us to monitor B–Z transitions under different conditions. The unique features of 2′F-modified DNA should thus be a valuable addition to existing techniques for specific detection of new Z-binding proteins and ligands.

## Graphical Abstract

**Graphical Abstract:**
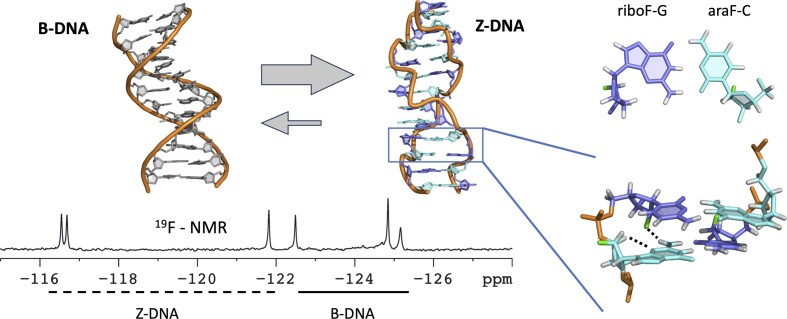

## Introduction

The left-handed Z-form has been shown to have an important role in several biological processes (1), including the regulation of gene expression (2,3), nucleosome positioning (4,5), and genetic instability associated with DNA damage and repair (6,7). As such, Z-form DNA has become an important therapeutic target (8,9). Moreover, multiple proteins associated with cancer and inflammation have been identified with high specificity for Z-DNA, including ADAR1, DLM1, PKZ, ZBTB43 and E3L (10–16). Today, ADAR, which is a double-stranded RNA editing protein, is being explored in the therapeutic space to correct disease-causing mutations in RNA (17–19). The p150 isoform of ADAR1 contains a Zα domain which recognizes and binds to Z-DNA and Z-RNA specifically (20–22). Hence, this structural feature may be exploited for improved ADAR1 editing of target RNA (18,19). Z-forms at the DNA and RNA levels have been associated with other pathogen-associated molecular patterns. For example, very recently, Z-DNA has been proposed as a structural component of the bacterial biofilm matrix (23), while Z-RNA has been linked to virus-induced cell necroptosis (24).

Despite their established biological relevance, Z-form duplexes are only stable *in vitro* under high ionic strength (i.e. 3–4 M NaCl) or in certain solvents (25). Under these conditions, the purines of Z-form favoring sequences (pyrimidine/purine repeats) exist in the North sugar pucker with a *syn N*-glycosidic orientation, thereby prompting Z-DNA into its characteristic zig-zag structure. Strategies to induce B-to-Z transitions near physiological conditions have relied on Z-DNA ligands (26,27) and nucleoside modifications (28). Most successful modified nucleosides have been guanosine *syn*-form stabilizers (29–34), many of which are not commercially available. Most of these modifications involve the introduction of bulky substituents at the C8 position of guanosine, which hinder the *anti*-conformation of the glycosidic angle and consequently destabilize the B-form. Meanwhile, the effect of sugar pucker on Z-form stability has been largely underexplored (35–37). Herein we report that commercially available, South-oriented 2′-deoxy-2′-fluoroarabinocytidine (2′F-araC, abbreviated as afC) and North-oriented 2′-deoxy-2′-fluororiboguanosine (2′F-riboG, abbreviated as rfG) stabilize Z-form duplexes that still bind to the Zα domain of ADAR1 p150. Importantly, the two modifications act in synergy leading to a dramatic change of conformational preference from B- to Z-form. The structural determination by NMR methods and state-of-the-art computational studies revealed key sequential sugar-base interactions that exclusively occur in the Z-form. These interactions are responsible for a comprehensive B→Z transition under low salt concentrations compatible with physiological conditions. The ^19^F-NMR spectra of these modified sequences exhibit chemical shift differences between the B-form and Z-form over an unprecedented range. This finding expands the possibilities for monitoring B/Z transitions and enables the conformationally specific detection of new Z-binding proteins and ligands.

## Materials and methods

### Oligonucleotide synthesis

The synthesis and characterization of 8-bromo-2′-deoxy-2′-fluoro-riboguanosine (N-ibu) and its phosphoramidite derivative are described in the Supplementary Data (Supplementary Methods). Oligonucleotide synthesis was performed on an ABI 3400 DNA synthesizer (Applied Biosystems) at 1 μmol scale on Unylinker (ChemGenes) CPG solid support. Phosphoramidite derivatives of N-acetyl-2′-deoxycytidine (dC), N2-isobutyryl-2′-deoxyguanosine, 2′-deoxy-2′-fluoroarabino cytidine, 2′-deoxy-2′-fluoro-riboguanosine, and 8-bromo-N2-isobutyryl-2′-deoxy-2′-fluoro-riboguanosine were used at 0.1 M concentration in acetonitrile. Coupling times were 200 s for dC, 300 s for dG, 600 s for 2′F-araC and 900 s for 2′F-riboG and 8-bromo-riboF-G. Cleavage of DMTr groups (including that of the 8-Br rFG amidite) was carried out with 3% trichloroacetic acid in dichloromethane for 95 seconds. Oligonucleotide deprotection and cleavage from the solid support were achieved using 30% aqueous ammonium hydroxide at 55°C for 16 h, except for sequences containing 8-bromo where room temperature and 24 h were used. Hexamer sequences were purified by anion exchange HPLC on a Waters 1525 instrument using a Source 15Q Resin column (11.5 cm × 3 cm). The aqueous buffer system consisted of Solution A (25% acetonitrile, 15 mM sodium acetate) and Solution B (0.5 M lithium perchlorate, 25% acetonitrile, 15 mM sodium acetate) (solution A) at a flow rate of 10 ml/min. The gradient was 0−100% lithium perchlorate over 50 min at 60°C. Under these conditions the desired peaks eluted at roughly 23–25 min. The purified oligonucleotides were desalted using Glen-pack desalt columns (Glen Research), and their masses were confirmed by high-resolution LC–MS.

### CD spectroscopy

CD spectra were recorded at 10 or 25°C on a Chirascan VX CD spectrometer equipped with a Peltier temperature controller. Each sample was prepared at 75 μM or 125 μM in either 10 mM sodium phosphate buffer (pH 7.0) with varying concentrations of NaCl (between 0 and 3 M), which are indicated in the text. For some samples, we observed that oligonucleotide solutions turned cloudy at 3 M NaCl. Each sample was annealed at 90°C and allowed to cool to room temperature before acquisitions. For each sample, three scans were accumulated over a wavelength range of 200–350 nm in a 0.1 cm path length cell. Parameters used included a time per point of 0.5 s, sampling of 0.5 s, and a bandwidth of 1 nm. The buffers alone were also scanned, and their spectra subtracted from the average scan for each respective sample. CD spectra were collected in units of millidegrees. Data were smoothed using the Savitzky–Golay function within the Chirascan graphing software.

### NMR spectroscopy

Samples for NMR experiments were dissolved (in Na^+^ form) in either D_2_O or 9:1 H_2_O/D_2_O, 10 mM sodium phosphate buffer. Oligonucleotide concentration for 2D experiments was 0.5 mM. The pH was adjusted right before performing the NMR experiments by adding aliquots of concentrated HCl or NaOH. pH was measured with a Mettler Toledo pH-meter equipped with a micro electrode. All NMR spectra were acquired on Bruker Neo Avance spectrometers operating at 600 and 800 MHz equipped with cryoprobes and processed with the TOPSPIN software. 2D NMR experiments for spectral assignment and acquirement of experimental constraints were recorded at *T* = 5ºC. A jump-and-return pulse sequence was employed to observe the rapidly exchanging protons in 1D H_2_O experiments. NOESY spectra in D_2_O and 9:1 H_2_O/D_2_O were acquired with mixing times of 150 and 250 ms. TOCSY spectra were recorded with the standard MLEV-17 spin-lock sequence and a mixing time of 80 ms. In most of the experiments in H_2_O, water suppression was achieved by including a WATERGATE module in the pulse sequence prior to acquisition. The spectral analysis program SPARKY was used for semiautomatic assignment of the NOESY cross-peaks and evaluation of the NOE intensities.

### NMR constraints

Qualitative distance constraints were obtained from NOE intensities. NOEs were classified as strong, medium, or weak, and distance constraints were set accordingly to 3, 4 or 5 Å. In addition to these experimentally derived constraints, hydrogen bond constrains for the base pairs were used. Target values for distances and angles related to hydrogen bonds were set to values obtained from crystallographic data in related structures. Force constants were 20 kcal/mol·Å^2^ for experimental distance constraints, and 30 kcal/mol·Å^2^ for hydrogen bond distance constraints. Due to the relatively broad linewidths of the sugar proton signals, J-coupling constants were not accurately measured but roughly estimated from H1’–H2’ and H1’vH2’ DQF-COSY cross-peaks. When these cross-peaks were consistent with deoxyribose conformations in South or South/East, sugar dihedral angles were constrained to the following target intervals: ν0(–36.5°, –6.5°); ν1(19.8°, 49.8°); ν2(–49.8°, –19.8°); ν3(6.5°, 36.5°) and ν4(–15.0°, 15.0°), with a force constant of 25 kcal/mol·rad. This is equivalent to loosely constraining the sugar pseudorotation phase angles (Ps) between 144° and 180°.

### Structural calculations

Structures were calculated with the SANDER module of the molecular dynamics package AMBER 18 (38). The coordinates of the control duplex in Z-form (3P4J) with the corresponding modifications to include afC and rfG residues were taken as starting points for the AMBER refinement, consisting of an annealing protocol in water, followed by trajectories of 500 ps each in which explicit solvent molecules were included and using the Particle Mesh Ewald method to evaluate long-range electrostatic interactions. Other details of the specific protocols used in these calculations have been described elsewhere (38). The BSC1 force field (39) was used to describe the DNA and the TIP3P model was used to simulate water molecules. Force-field parameters for fluorinated sugars were obtained as described below. Analysis of the final structures was carried out with the program MOLMOL (40) and X3DNA (41). The conformation with the lowest AMBER total energy is taken as the best structure. Coordinates are deposited in the PDB data bank (codes for **aFC3**, **rFG3**, and **FC3-rFG3** structures are 8QDU, 8QE4 and 8QOR, respectively).

### Systems set-up

Eight different sequences, i.e. (aFC-rFG)_3_, (aFC-rFG)_10_, (aFC)_3_, (aFC)_10_, (rFG)_3_, (rFG)_10_, d(CG)_10_ and (aFC-rFG)_10_, were assembled in B and Z conformations, for a total of 16 independent systems. Initial coordinates for the oligonucleotides in Z-conformation were obtained from the NMR models. The rest of the initial coordinates were generated either with the AmberTools NAB package v.16 (42) for the B-conformers or with Web x3DNA v.2.0 for the Z-conformations. As these packages generate just canonical RNA or DNA, DNA structures were generated, and the corresponding hydrogen atoms were substituted with fluorine in the case of aFC and rFG.

Oligomers and long strands were solvated with cubic boxes of TIP3P water molecules with a minimum distance of 15 Å between any atom in the chains and the edge of the box. Then, either 10 or 38 K^+^ ions were added to neutralize the systems (depending on whether they were 6 or 20 bp long duplex systems). In no case were more ions added, to simulate the experimental ionic strength of 0 M. The 6 bp duplexes contained between 20k and 25k atoms while the longer duplex systems contained between 120k and 150k atoms.

Water molecules were described by the TIP3P force field, while potassium cations were modeled by the Joung-Cheatham parameters (43) for monovalent ions. Nucleic acids were treated with the ParmBSC1 force field (39), adapted to the fluorinated sugars (where appropriate). New parameters for the puckering description were generated for 2′-fluorinated furanoses by fitting the potential energy profile for the ν2 torsion angle rotation. Potential energies were obtained by single point energy calculations employing the Møller–Plesset perturbation theory of second order (MP2) (44) with 6–311++G** basis set. Previous optimizations were carried out using the hybrid exchange-correlation Becke, three-parameter, Lee–Yang–Parr (B3LYP) functional (45) and 6–311++G** basis set. The DFT functional was supplemented with the D3 version of Grimme's dispersion (46) with Becke-Johnson damping (GD3BJ) (47). Water solvation was accounted using the SMD version (48) for the integral equation formalism variant of the polarizable continuum model (IEF-PCM) (49). Abovementioned calculations were performed in Gaussian 16 Rev. B.01 software (50). Force-field parameters for the modified nucleotides are available from the authors.

Global setup of the systems and the generation of the topology files were carried out with the AmberTools tLEaP package v.16 (42).

### Classical molecular dynamics

All molecular dynamics (MD) simulations were performed with the following sequence step protocol: (i) A minimization is performed using the conjugate gradient algorithm with a convergence criterion of 0.05 kcal·mol^−1^ or a maximum of 50k optimization steps. (ii) System is allowed to reach a stable energy distribution by a 1 ns 100 K NVT run before coupling the barostat. (iii) System is heated gradually in three 1 ns sequential NPT simulations from 100 K to 298.15 K, with a constant pressure of 1 bar. Finally, (iv) 250 ns-long NPT MD simulation is performed at 298.15 K and 1 bar. The first 100 ns of all simulations were discarded as the equilibration stage.

The cutoff limit for short-range interactions was 12 Å whereas a Particle Mesh Ewald (PME) model (51) was used to thread the long-range interactions. Temperature control was performed using Langevin dynamics thermostat (52) with a 0.5 ps^−1^ collision frequency in the production stage and 3.0 ps^−1^ in the heating steps. Pressure control was performed with Berendsen barostat (53) with a relaxation time of 0.5 ps in the production stage and 3.0 ps in the heating steps. In all stages, SHAKE algorithm (54) was used to constrain light-heavy atoms vibrations allowing an integration step of 2 fs. No additional restraints were used in any case. The Verlet algorithm was employed to propagate the system.

All classical calculations were run in the AMBER v.20 GPU software version (PMEMD) (42,55) and most of the analyses were performed using the AmberTools CPPTRAJ package v.16 (42).

### Quantum mechanical calculations

Representative structures were selected from the oligonucleotides’ MD trajectories using the clustering algorithm of fast search and find of density peaks (56). The root-mean-square deviation (RMSD) was used as the similarity criterion. Then, the centroids of the most populated clusters were classically optimized using the conjugate gradient algorithm with a convergence criterion of 0.05 kcal·mol^−1^ or a maximum of 50k optimization steps. From the optimized structures two base pairs were extracted and the O5′ and O3′ groups were capped with hydrogens as hydroxyl groups.

Then, reduced models were optimized with the Berny algorithm (57) implemented in Gaussian 16 Rev. B.01 software. The electronic structure was solved using B3LYP-GD3BJ/6–311++G** with an SCRF = SMD solvent treatment. In all cases, harmonic restraints were applied to the initial structures with force constants of 5 kcal·mol^−1^·Å^−2^ to avoid structural artifacts induced by the reduced models.

Topological analyses of the electron density (also known as AIM analyses) (58,59) were performed on the reduced models to locate the critical points (CPs). Main CPs properties were then decomposed as orbital contributions. Analyses of the CPs were complemented with visual studies of the non-covalent interactions based on the reduced electron density gradient (RDG/NCI analyses) (60). All CP/AIM and RDG/NCI analyses were carried out using the Multiwfn v.3.8 electronic wave function analysis software (61).

### Electrophoretic mobility shift assays (EMSA)

The peptide sequence of the Zα domain of the p150 isoform of ADAR1 was purchased from BIOMATIK, consisting of ADAR1p150 amino acid residues 122–199 with a GST tag at the N-terminus (62).

The shift assays were performed using 10% nondenaturing acrylamide/bis-acrylamide (19:1) gel in 1× TBE buffer solution. A 5 μM solution of each duplex tested was annealed in 10 mM sodium phosphate buffer (pH 7). A stock solution of the peptide (4 nM) was prepared in a buffer containing 20 mM sodium phosphate (pH 7), 20 mM NaF and 0.5 mM DTT. The peptide was added to the samples in the described ratios, followed by an incubation period of 40 min at room temperature. Glycerol was added to each sample prior to loading onto the gels, with a final glycerol concentration of 15%.

Electrophoresis was performed at 14 V·cm^−1^ for 1.5 h in 1× TBE running buffer at 5 °C. Subsequently, the gels were stained with SYBR Gold, visualized using Bio-Rad Gel Doc XR and the images were processed with the Image Lab software package.

## Results and discussion

### afC and rfG promote Z-DNA formation

**Figure 1.**
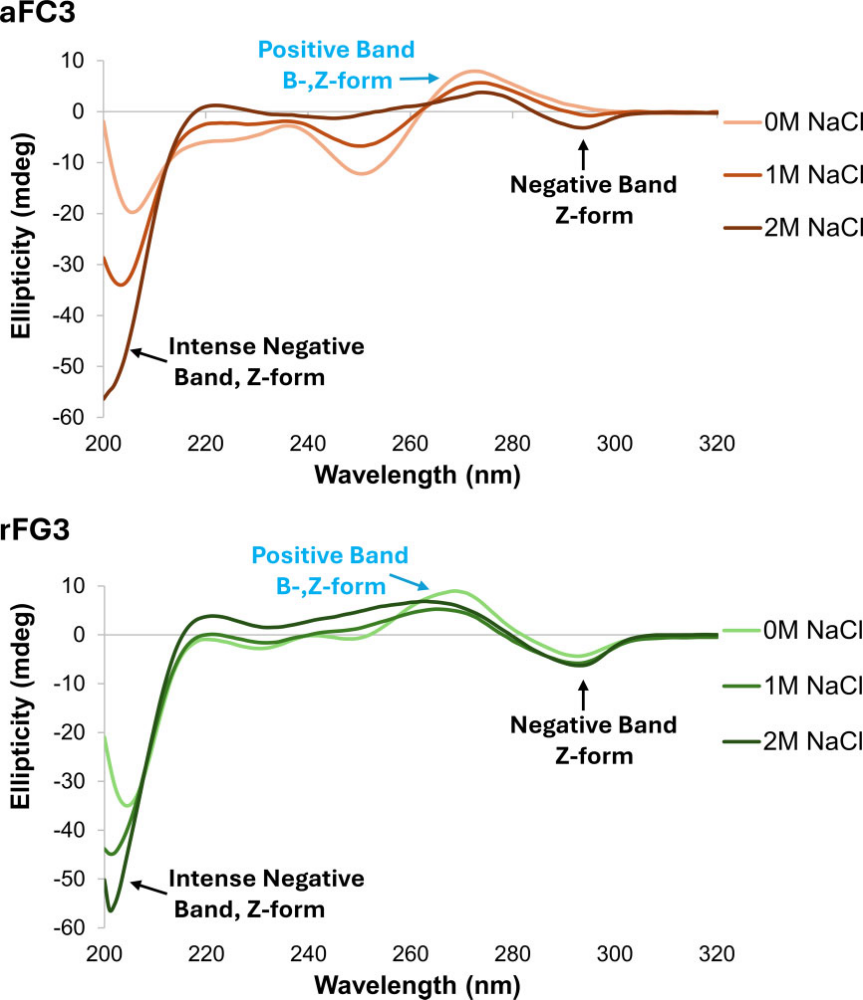
CD spectra of **aFC3** (top) and **rFG3** (bottom) at different NaCl concentrations. Experimental conditions: 10 mM NaPi buffer, pH 7.0, *T* = 10 °C, [oligonucleotide] = 75 μM.

First, we analyzed a series of four (CG)_3_ oligonucleotide sequences with incremental dC to afC substitutions (Table 1) by CD and ^19^F NMR spectroscopy. Z-form duplex formation can be detected through CD spectroscopy by monitoring the emergence and intensity of the negative band at 295 nm, or the intensity of the negative band at 200 nm. (63) With each additional afC substitution, we observe that the intensification of the negative band at 295 nm occurs at lower salt concentrations (Figure 1 and Supplementary Figure S1), suggesting that afC substitutions incrementally confer stability to the Z-form duplex. Midpoints for the salt induced transitions could be estimated from the intensity of the negative band, and are around 2.6 M for **CTRL**, 2.0 M for **aFC1**, 1.6 M for **aFC2**, and 1.0 M for **aFC3**.

**Table 1.** (CG)_3_ Oligonucleotide sequences studied^a^

Code	Sequence (5′-3′)					
**CTRL**	C	G	C	G	C	G
**aFC1**	C	G	afC	G	C	G
**aFC2**	C	G	afC	G	afC	G
**aFC3**	afC	G	afC	G	afC	G
**rFG3**	C	rfG	C	rfG	C	rfG
**aFC3rFG1**	afC	G	afC	rfG	afC	G
**aFC3rFG2**	afC	rfG	afC	rfG	afC	G
**aFC3rFG3**	afC	rfG	afC	rfG	afC	rfG

^a^C and G = dC and dG respectively; afC = 2′F-araC; rfG = 2′F-riboG.

**Figure 2.**
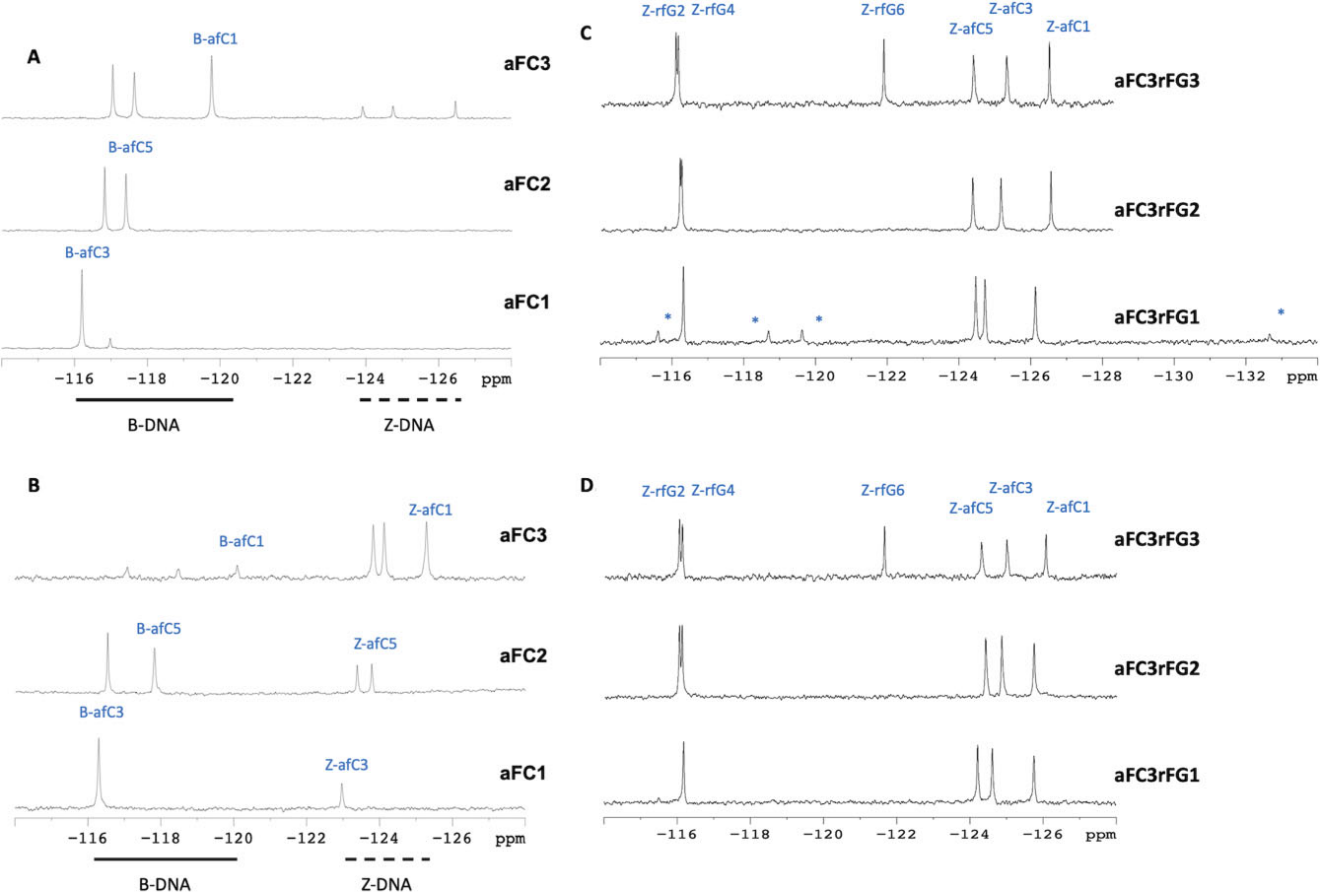
^19^F-NMR spectra of **aFC1**, **aFC2** and **aFC3** at 0 M (**A**) and 2 M NaCl (**B**). ^19^F-NMR spectra of **aFC3rFG1**, **aFC3rFG2** and **aFC3rFG3** at 0 M (**C**) and 1 M NaCl (**D**). Experimental conditions: 10 mM sodium phosphate buffer, pH 7.0, *T* = 25°C, [oligonucleotide] = 0.5 mM. *Indicates signals from a minor species only observed at low salt concentration in **aFC3rFG1**.

**Figure 3.**
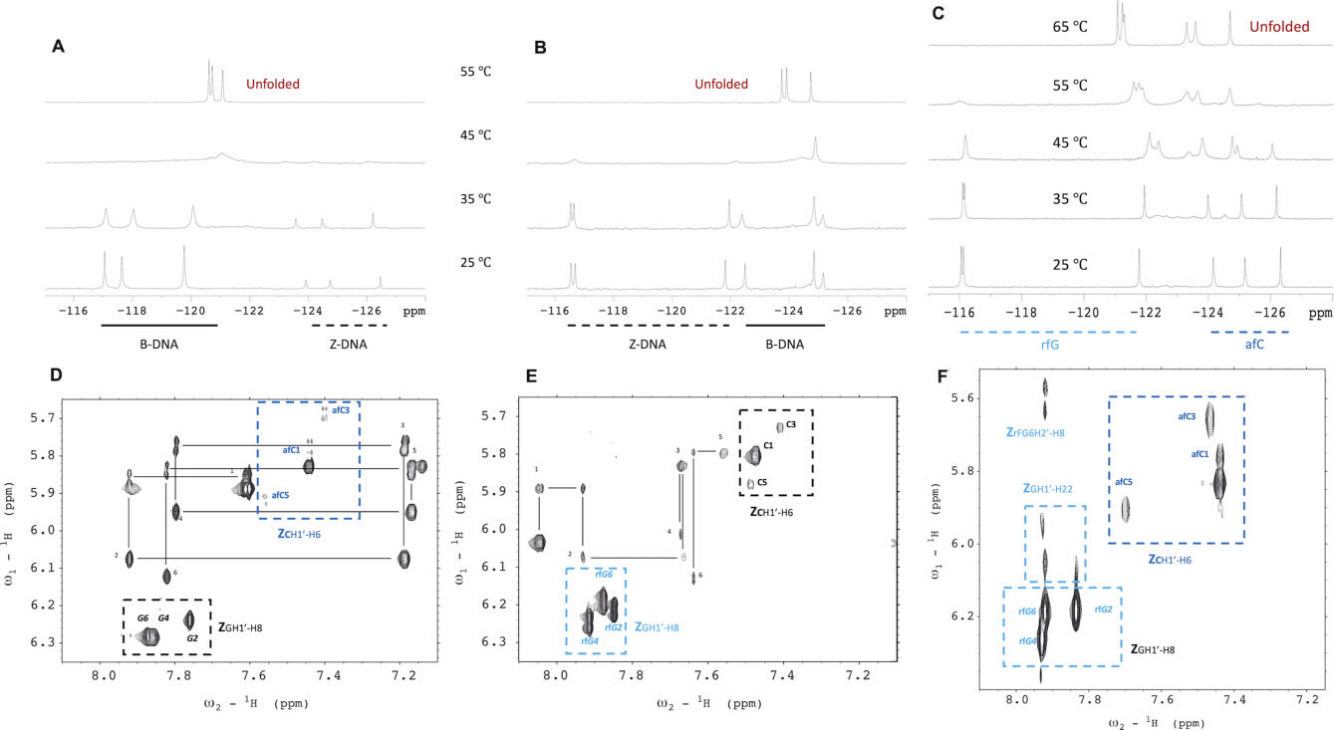
^19^F-NMR spectra of (**A**) **aFC3**, (**B**) **rFG3** and (**C**) **aFC3rFG3** at different temperatures. Population of Z-form species at *T* = 25ºC estimated from the relative signal intensities are 20%, 60% and 100% for **aFC3**, **rFG3** and **aFC3rFG3** respectively. H1′-aromatic region of the NOESY spectra (*τ*_m_ = 250 ms) of (**D**) **aFC3** (*T* = 15 °C), (**E**) **rFG3** (*T* = 5 °C), (**F**) **aFC3rFG3** (*T* = 25 °C) [buffer conditions: 10 mM NaPi, pH 7.0].

Results from the ^19^F NMR spectra (Figures 2 and 3) agree well with those from CD spectroscopy. The ^19^F signals are very well-dispersed, with the number of signals being consistent with slow equilibria (≥ms) in the NMR time scale between the B and Z forms. ^19^F signals can be easily assigned to B or Z forms by taking advantage of their dependency with salt concentration (Figure 2). B- and Z-form signals appear in distinct regions of the ^19^F NMR spectra, from –116 to –120 ppm for the B-form, and from –124 to –126 ppm for the Z-form. This chemical shift difference (6–8 ppm) is one order of magnitude larger than those previously observed in DNA duplexes with fluorine substituted nucleobases (64–66). The B and Z form signals become broad and disappear close to the melting temperature, indicating that the equilibria with the denatured oligonucleotide are intermediate in the ^19^F chemical shift time scale (Figure 3). Then, sharp signals corresponding to unhybridized oligonucleotides appear again at 55–65°C. As expected, they are not so well-dispersed, as they appear in a narrow range of the spectra, between –120 and –121 ppm. However, they are perfectly distinguishable from B and Z form signals, indicating that afC is an excellent probe to detect DNA conformational state by NMR. Moreover, the population of the different species can be readily quantified by evaluating the relative intensity of their corresponding ^19^F signals (Supplementary Figure S2). Although aggregation processes are apparent in the NMR spectra at high salt concentrations, the relative ^19^F signal intensities of B and Z species are consistent with the populations obtained from CD data.

We also explored the effect of rfG by substituting all dG residues of **CTRL**, as in sequence **rFG3** (Figure 1). As in the case of afC substitutions, rfG ^19^F signals from B and Z species appear in different regions. However they are shifted in opposite directions: rfG signals are more downfield when rfG is in a Z-form environment, while they are more upfield when rfG is in a B-form environment (Figure 3B). This might be due to changes in syn/anti conformation of the N-glycosidic bond. Moreover, we observe that sequence **rFG3** has a larger proportion of Z-duplex at 25°C and low salt in comparison to sequence **aFC3** (60% versus 20%, respectively). At 55°C, the duplexes of both sequences are unfolded, with the signals from afC being more downfield than those from rfG.

### Combining afC and rfG in the same strand

**Figure 4.**
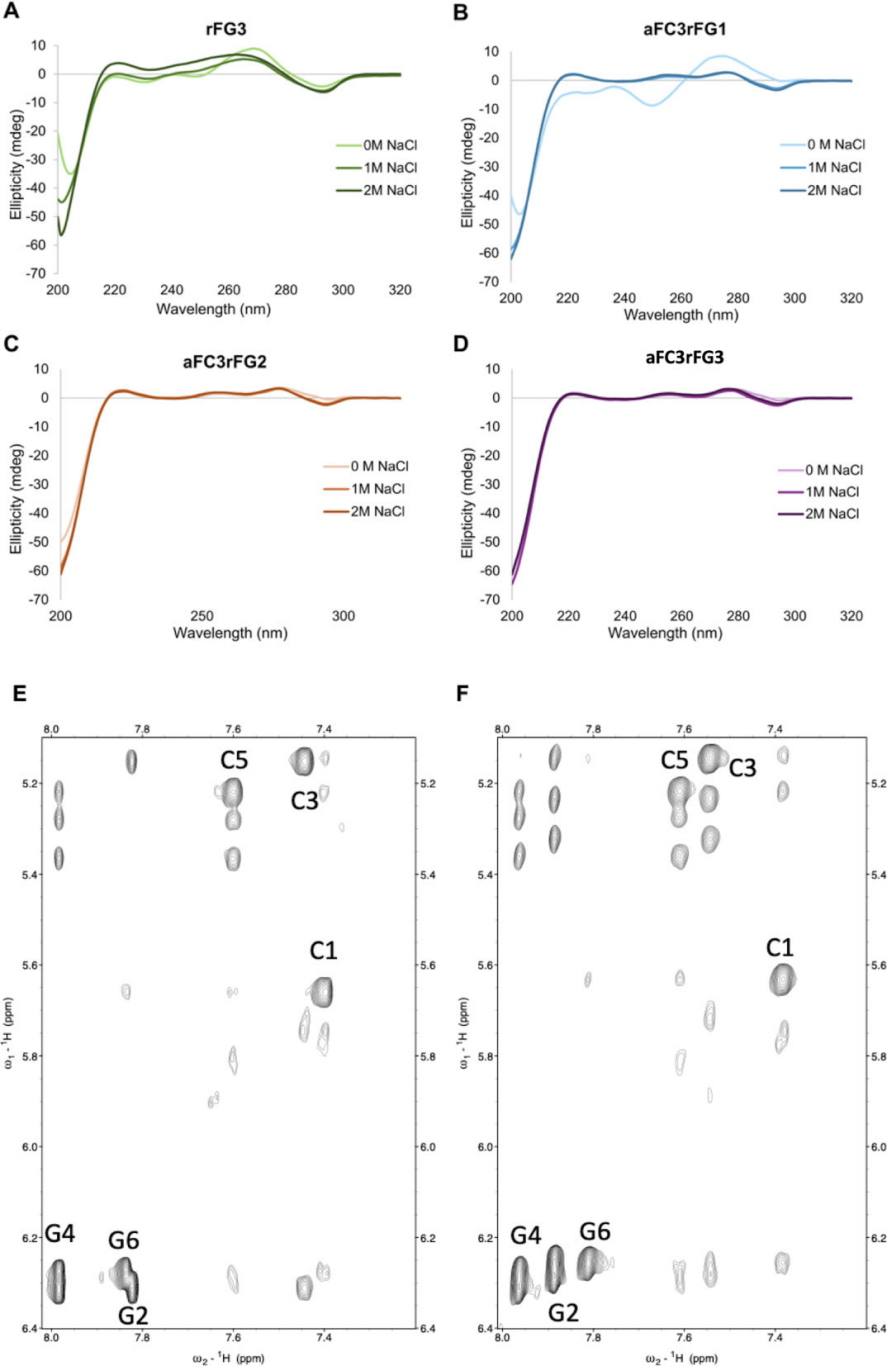
CD spectra of **rFG3** (**A**), **aFC3rFG1** (**B**), **aFC3rFG2** (**C**) and **aFC3rFG3** (**D**) at increasing NaCl concentrations; [oligonucleotide] = 75 μM. Experimental conditions: 10 mM sodium phosphate buffer, pH 7.0, *T* = 10 °C. Some CD spectra at high NaCl concentration are omitted due to sample precipitation. (**E**) Regions of the 2D NOESY spectra (250 ms mixing time, *T* = 5°C) of **aFC3rFG1** (**E**) and **aFC3rFG2** (**F**). Guanine H1’–H8 intraresidual cross-peaks are labelled and exhibit a high intensity, comparable to cytosines H5-H6 NOEs (also labelled), indicating that guanine (rfG and dG) glycosidic angles are all in *syn* [buffer conditions: 10 mM NaPi, 1 M NaCl, pH 7].

Considering the propensity of afC and rfG to promote the Z-form, we aimed to investigate the combined effect of these two substitutions. Thus, we prepared sequences which combine afC and rfG in the same strand (Table 1). The number of signals in the ^19^F NMR spectra indicate the presence of a single species for **aFC3rFG2** and **aFC3rFG3** (Figure 2C, D). A minor component is observed in the case of **aFC3rFG1** only at low salt conditions. Z-form formation is clearly established by the upfield and downfield ^19^F NMR shifts of afC and rfG, respectively, and confirmed by the negative bands at 200 and 295 nm in the CD spectra (Figure 4). In addition, the observation of very strong H8-H1’ cross-peaks in the 2D NOESY spectra indicates that all purines (rfG and dG) are in syn conformation, confirming the full transition to the Z-form (Figure 4), and thereby and demonstrating a synergistic effect of afC and rfG, when combined in the same strand. The effect is so dramatic that Z-form duplexes are the only species observed in the NMR spectra of samples **aFC3rFG2** and **aFC3rFG3** in 10 mM sodium phosphate buffer and neutral pH (Figure 2C).

Similar to sequences **aFC3** and **rFG3**, the fluorine signals corresponding to afC and rfG in the Z form are located within the range of –123 to –127 ppm and –116 to –122 ppm, respectively. The effect of temperature in the fully modified sequence **aFC3rFG3** is analogous to that observed previously, characterized by significant signal broadening at temperatures near the melting transition (Figure 3C). Interestingly, no signals corresponding to the B-form are observed at any temperature.

It should be noted that the CD spectrum of **aFC3rFG1** at 0 M NaCl concentration suggests the co-existence of significant populations of both left- and right-handed duplex populations, with ellipticity value at 295 nm approaching 0, a positive band at 280 nm, and an intense negative band at 200 nm (Figure 4). Meanwhile, the ^19^F NMR spectrum clearly indicated that the major species is the Z-form, with an estimated population of ca. 85% from the relative signal intensities (Figure 2). This minor discrepancy is most probably due to differences in oligonucleotide concentrations, whereby higher concentrations used in NMR experiments are conducive to larger populations of Z-form duplexes.

### Combining afC and rfG in different strands

The ^19^F-NMR spectra obtained from sequences **aFC3**, **rFG3** and **aFC3**+ **rFG3** (a 1:1 mixture of **aFC3** and **rFG3**) are shown in Supplementary Figure S3. Signals corresponding to the B and Z forms of the two homoduplexes (**aFC3:aFC3** and **rFG3:rFG3**) can be distinguished by their presence in the pure **aFC3** or **rFG3** samples. The emergence of additional signals indicates the presence of new species corresponding to the mixed hybridization of the two fluorinated sequences (**aFC3**:**rFG3**) (black arrows in Supplementary Figure S3). Under low salt concentration, the number of additional signals is consistent with the presence of B- and Z-form species. However, upon increasing the ionic strength to 2 M NaCl, the number of signals decreases, revealing only those associated with the Z conformation of the two homoduplexes and the hybrid (indicated with black dashed arrows in Supplementary Figure S3). By analyzing the relative intensities of the fluorine signals in these spectra, we were able to estimate the populations corresponding to each species within the **aFC3**+ **rFG3** mixture. Under 0 M NaCl conditions, the B forms predominate in the mixture and, upon increasing the ionic strength, the populations of the Z-form in the **rFG3:rFG3** homoduplex and the **aFC3:rFG3** heteroduplex remain very similar. This led us to conclude that afC substitutions on one strand and rfG substitutions on the complementary strand provide no additive effects under the conditions studied.

### NMR assignment and structural calculation

To get insight into the stabilization imparted to the Z-duplexes by the 2′F-modification, we undertook the structural determination of sequences **aFC3**, **rFG3** and **aFC3rFG3** under low salt conditions. With this aim, we performed the complete assignment of the ^1^H and ^19^F NMR spectra (Supplementary Tables S1–S3).

**Figure 5.**
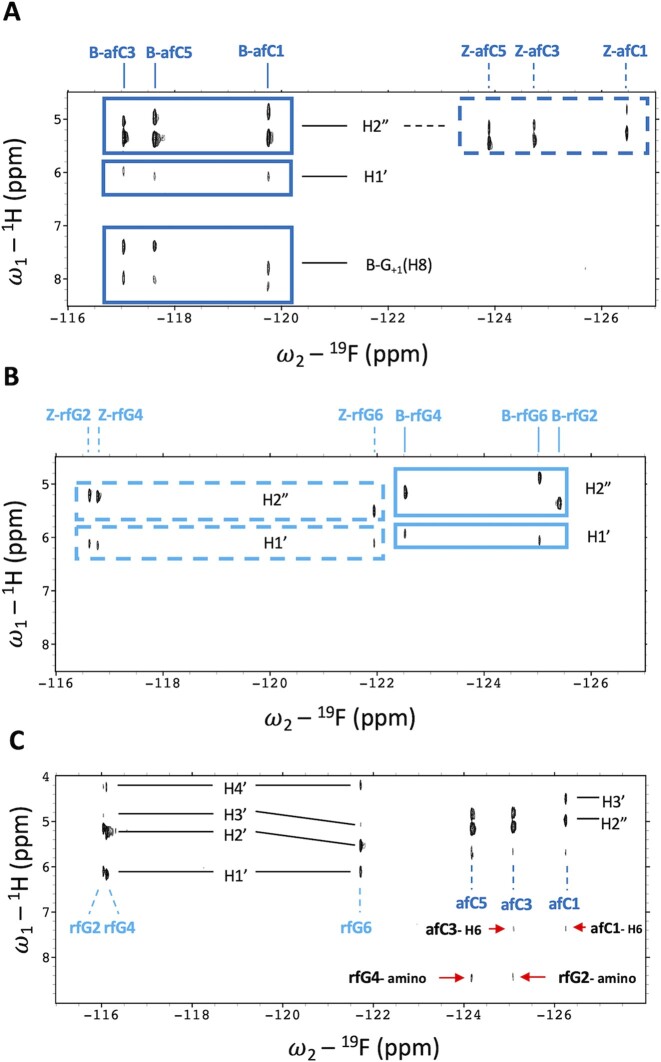
^19^F-^1^H HOESY spectra of (**A**) **aFC3**, (**B**) **rFG3** and (**C**) **aFC3rFG3**. Key fluorine–aromatic proton cross-peaks in **aFC3rFG3** are indicated with red arrows [buffer conditions: 10 mM NaPi, 100% D_2_O, pH 7.0, *T* = 25°C].

Exchangeable proton spectra confirmed the formation of G:C Watson–Crick base pairs (Supplementary Figure S4). 2D spectra (Figure 3D–F) showed the co-existence of two species in **aFC3** and **rFG3**, and a single species in **aFC3rFG3**. Despite the coexistence of B- and Z-forms in **aFC3** and **rFG3**, the good signal dispersion allowed the complete assignment of both species. The B-forms could be assigned following standard pathways for right-handed helices, as shown in Figure 3D and E. In **aFC3**, **rFG3**, and **aFC3rFG3**, the ^1^H resonances of the Z-forms could be distinguished by the characteristic strong H1′-H8 NOE of the *syn* guanines. Then, sequential assignments could be followed in the H2′/2″-aromatic region (see Supplementary Figure S5). ^19^F signals were assigned through the intense cross-peaks with the protons of their own sugar in the heteronuclear ^19^F-^1^H HOESY spectra (Figure 5).

No ^19^F-^1^H cross-peaks with aromatic protons were observed for rfG residues in either the B- or Z-form. However, intra-residual and sequential ^19^F-aromatic proton heteronuclear Overhauser effects (HOEs) were clearly visible for afC residues in the B-form. Most interestingly, a singular pattern of ^19^F-^1^H HOESY cross-peaks is observed in afC residues of **aFC3rFG3**, involving both intra-residual 2′F-H6 cross-peaks and sequential cross-peaks between 2′F and an amino proton of the 5′-neighbouring guanine (Figure 5C).

Qualitative estimation of ^1^H–^1^H J-coupling constants was obtained from DQF-COSY experiments (Supplementary Figure S6). In **aFC3rFG3**, the low J_1′2″_ value of the modified cytosines suggests a South-East (C2′–O4′ endo) tendency, in contrast to the south orientation in **aFC3** and **rFG3** duplexes. On the other hand, the guanines are clearly in North (C3′-endo), as indicated by the absence of H1′-H2′ DQF-COSY cross-peaks.

The three-dimensional structures of the Z-forms were calculated based on NMR data by following protocols described in the Experimental Methods section. Statistical analysis of the final structures including the number of distance and angular constraints are shown in Table S4. In all cases, the structures are overall well-defined with RMSD deviations below 1 Å. However, the **aFC3rFG3** duplex is the best defined, probably due to the larger number of experimental constraints, a consequence of the Z form being the only species under these conditions.

### Description of the Z duplexes

**Figure 6.**
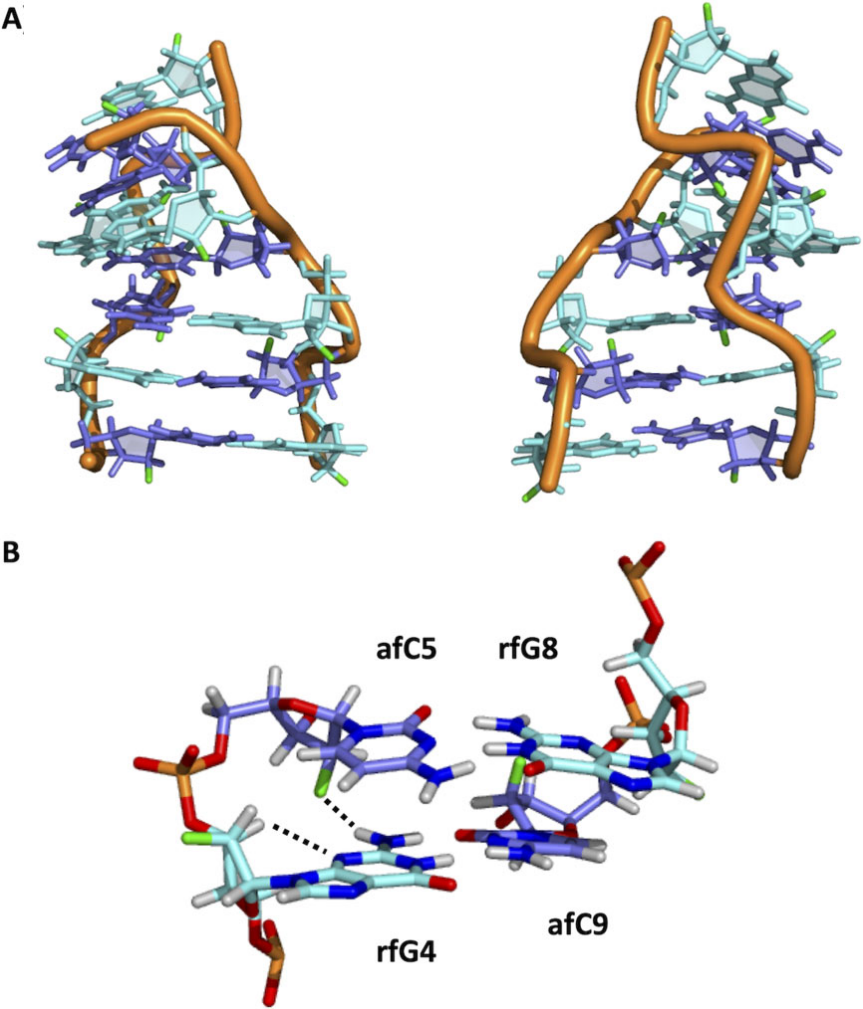
(**A**) Two views of the solution structure **aFC3rFG3**. (**B**) Detail of key close contacts: sequential 2′F(afC)..amino(rfG) and intraresidual H3′..N of rfGs.

The resulting structures of the Z-forms of **aFC3**, **rFG3** and **aFC3rFG3** are shown in Figure 6 and Supplementary Figure S7. In all cases, structures share the general features of Z-DNA. Glycosidic angles are all very similar to those observed in unmodified Z-DNA. Cytidine sugars are always in the South domain (P_s_ between 130º and 180º), whereas rfGs are North and dGs in **aFC3** are generally South-East. Geometrical parameters are shown in Tables S5-S7. Minor groove size in **aFC3rFG3** is similar to standard Z-DNA. However, **aFC3** and **rFG3** exhibit broader and narrower minor groove size, respectively.

Whereas rfG fluorine atoms are mainly exposed in the surface, those of afCs point towards the narrow minor groove. In the case of **aFC3rFG3**, the fluorines are located very close to the amino groups of their 5′-neighbouring rfGs (Figure 6B). This is totally consistent with the HOEs observed (see Figure 5C). Interestingly, the distance (F2′|afC)-(H21|dG) in **aFC3** is considerably larger (>1 Å).

### Molecular dynamics simulations

To gain a better understanding of the significance of these structural features in the relative stabilities of B- and Z- forms, we employed a variety of *state-of-the art* theoretical methods. We conducted extensive molecular dynamics simulation starting from the NMR structures, as well as from a control 6-mer unmodified duplex DNA. The B- and Z- conformations of the **aFC3** and **rFG3** systems were stable throughout the simulation, as reflected in a RMSD of the sampled structures that diverged by 1–1.5 Å from the reference conformations (Supplementary Figure S8).

On the contrary, **aFC3rFG3** quickly deviates from the B-conformation, and remains near the Z-form. Similarly, the control DNA duplex displayed a preference for the B-form as expected (Supplementary Figure S8).

**Figure 7.**
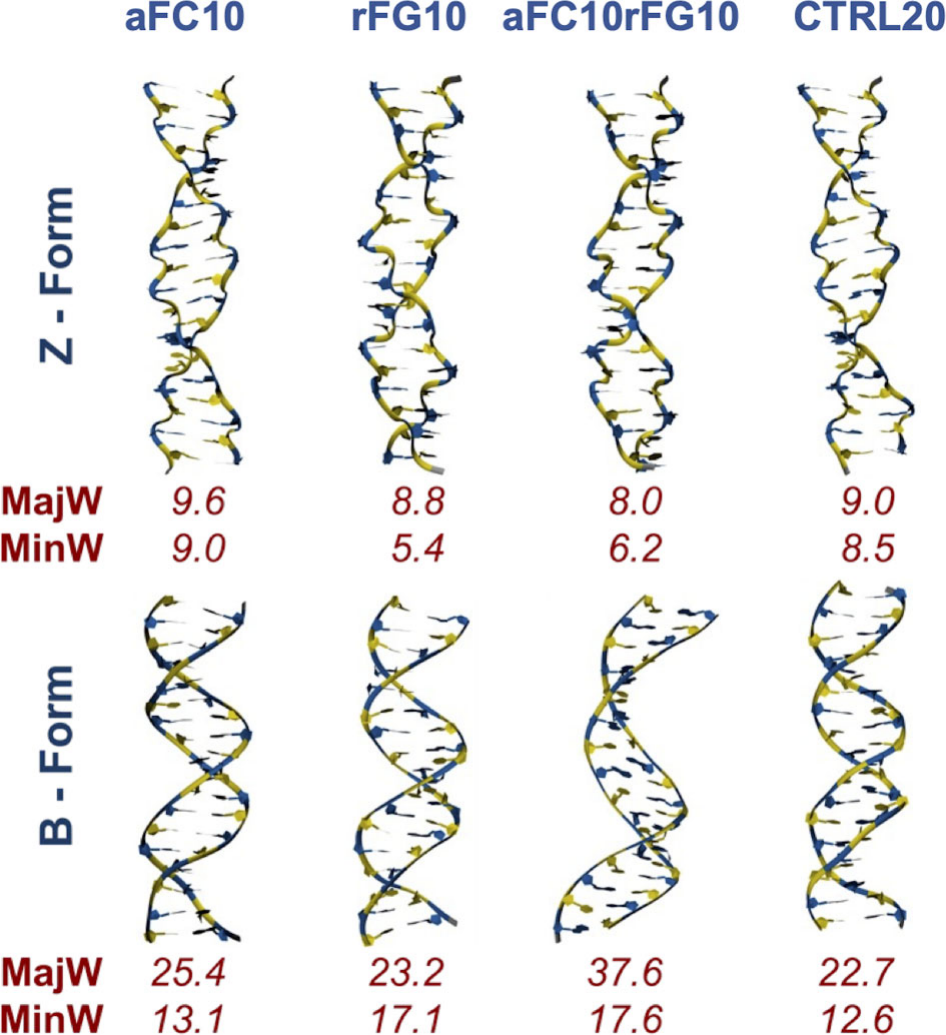
Representative structures of the 150 ns MD trajectories for (aFC-dG)_10_ (**aFC10**), (dC-rfG)_10_ (**rFG10**), (afC-rfG)_10_ (**aFC10rFG10**) and d(GC)_10_ (**CTRL20**) sequences starting from canonical B- (top) and Z- (bottom) conformations. Strands are represented in New Ribbons with dCs and afCs colored in yellow, and dGs and rfGs in blue. Major (MajW) and minor (MinW) groove widths are reported in Å below each corresponding structure.

It is worth noting the good agreement between these unbiased simulations and spectroscopic data, even in cases where an unprecedented stabilization of the Z-form is detected. However, the limited size of the duplexes may raise questions about how general these results are, and their applicability to longer oligonucleotides. To address this concern, we performed simulations of four 20 bp duplexes, namely, (dCdG)_10_, (afCdG)_10_, (dCrfG)_10_, and (afCrfG)_10_, denoted respectively as **CTRL20**, **aFC10**, **rFG10** and **aFC10rFG10** (Figure 7). For each case, trajectories started from B- and Z- conformations and deviations from the canonical B/Z structures were utilized as indicators of the relative B/Z stability of the duplex. The control DNA consistently showed a preference for the B-conformation and moved apart from the canonical Z-form, as observed experimentally (see Figure 7, and Supplementary Figures S9 and S10). The opposite situation is observed in **aFC10rFG10**, which is unstable in the B-form while remaining very close to the canonical Z-form (see Figure 7, and Supplementary Figures S9 and S10), again confirming the spectroscopic data obtained in shorter oligos.

The situation of **rFG10** and **aFC10** were intermediate between these two extreme cases. **rFG10** exhibits a greater preference for the Z- than the B-conformation, while **aFC10** displays slightly more stability in the B- than in the Z-form.

However, alternative conformations were also observed in both cases, as experimentally found in shorter duplexes. In summary, MD simulations allow us to extend experimental observations derived from NMR and CD studies on small oligonucleotides, defining a general (length-independent) relative Z abundance at low ionic strength: **CTRL20**<**aFC10** < **rFG10** < **aFC10rFG10**.

### Quantum mechanical calculations

**Figure 8.**
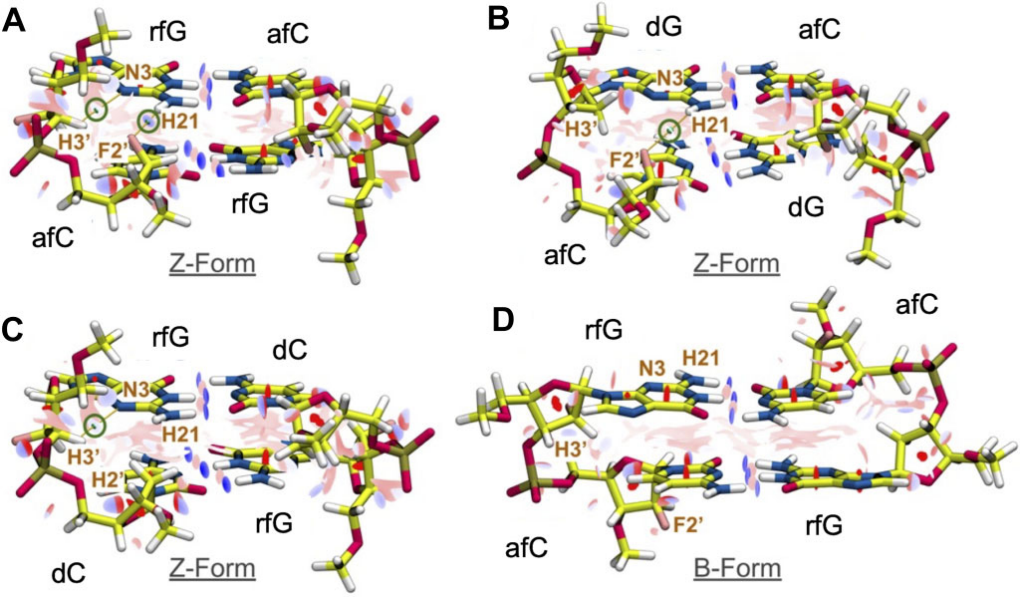
Reduced models of (**A**) aFC:rfG, (**B**) afC:dG, (**C**) rfG:dC base pairs in a Z-duplex and (**D**) a hypothetical afC:rFG base pair in a B-duplex system. NCI visual studio is represented (isosurface value *s* = 0.05) in red shades (repulsive interactions) and blue shades (attractive interactions). Relevant bond critical points are emphasized with green circles and atoms connected through each critical point are joined with orange paths. Critical points are numerically characterized in Supplementary Table S9.

The dramatic impact of fluorinated sugars on B- versus Z-conformational equilibrium is quite surprising, as the Z-form of DNA is known to be stable only under very high ionic strength conditions. The difference is so dramatic that it should imply the formation of some strong contacts mediated by the fluorine atom. To investigate this point, reduced models were extracted from trajectories, re-optimized at the QM level, and used to determine the associated wavefunctions (see Experimental Methods), from which topological analyses of the electron distributions were done. The study provides the critical points (CP) which are informative on individual non-bonded interactions (58,59,67–71). CP analyses were complemented with visual studies of the non-covalent interactions based on the reduced electron density gradient (RDG/NCI) (results are shown in Figure 8A–D) (60). A comparison of the **aFC10rFG10** models revealed two main interactions that stabilize specifically the Z- over the B-form: one between the F2′ atom of afC and the amino group of the sequential rfG, and another between the F-polarized H3′ of afC and the electron pair at N3 of rfG (see Figure 8A). The combined effect of both interactions synergistically promotes the Z- over the B-conformation in duplexes containing both afC and rfG. The (F2′|afC)-(H21|rfG) interaction is especially strong as indicated by an electron density (ρ(r)) of 2.04E-2 a.u. at the CP, compatible with a hydrogen bond (72), while (H3′|rFG)-(N3|rFG) interaction shows a non-negligible *ρ(****r****)* at the CP of 7.12E-3 a.u., more consistent with a moderate electrostatic interaction (see Supplementary Table S9) (72).

A (H3′|rfG)-(N3|rfG) interaction is also present in **rFG3** (*ρ(****r****)* of 7.66E-3 a.u.) consistent with the Z-form dominance observed for this duplex. However, without the (F2′|afC)–(H21|rfG) H-bond, experiments point out that this moderate interaction allows a fraction of the population to be promoted into the B-conformation. More interesting is the severe weakening of the hydrogen bond in the aFC models. The sequential (F2′|afC)–(H21|rfG) atomic distance average in the MD simulations varies from 2.21 Å in **aFC10rFG10** to 2.82 Å in **aFC10** (see Supplementary Table S9). This distance elongation drops the intensity of the interaction observed in the reduced model by almost an order of magnitude (*ρ(****r****)* of 2.59E-3 a.u.), explaining why duplexes with only afC lose the Z-form dominance, and most importantly, why the synergic effect between afC and rfG is not observed when the substitutions are not located in the same strand.

### 2′F-modificacions are compatible with C8 bulky substitutions

Classical nucleoside modifications inducing Z-DNA formation involve bulky substitutions at the guanine C8 position that disfavor the *anti* conformation, thereby destabilizing the B-form. To explore whether this strategy is compatible with our 2′F-sugar modifications, we synthesized the modified phosphoramidite analogue of 8-bromo-2′-deoxy-2′-fluoro-riboguanosine (8-Br-rfG, see the Supporting Information) and incorporated into the control sequence. Indeed, based on NMR and CD spectroscopy data (Supplementary Figure S11), we find that a single 8-Br-rfG substitution per strand is sufficient to stabilize the Z-form exclusively in 10 mM sodium phosphate buffer. This strategy harnesses the ^19^F-NMR based sensing capability of the rfG modification, while surpassing previous efforts at stabilizing Z-form duplexes with a minimal number of modified nucleosides.

### C/G sequences with a TA interruption site adopt Z-form duplexes

Interruption sites composed of T and/or A bases are known to disrupt Z-form duplex formation in (CG)_*n*_ sequences (64,73,74). To test the ability of 2′-fluorinated nucleosides to overcome the destabilization imparted by interruption sites, we designed a new pair of sequences, **CTRL-TA** and **FLUORO-TA** (Table 2) and characterized them by CD and NMR spectroscopy.

**Table 2.** Additional sequences used in NMR and EMSA studies^a^

Code	Sequence (5′-3′)					
**CTRL-TA**	C	G	T	A	C	G
**FLUORO-TA**	afC	rfG	afT	rfA	afC	rfG
**G_3_C_3_**	G	G	G	C	C	C
**FLUORO-G_3_C_3_**	rfG	rfG	rfG	afC	afC	afC

^a^C, G, T, A = dC, dG, dT, dA respectively; afC, afT = 2′F-araC, 2′F-araT, respectively; rfG, rfA = 2′F-riboG, 2′F-riboA, respectively.

As expected, **CTRL-TA** could not adopt Z-form character regardless of the NaCl concentration of the sample (Supplementary Figure S12). Meanwhile, **FLUORO-TA** shows clear intensification of the Z-form bands at 205 and 295 nm in the CD spectrum (125 μM). ^19^F NMR spectra are consistent with the formation of a single species with as observed in **aFC3rFG3**, although with a much lower thermal stability (Supplementary Figure S13B). This lower stability is clearly observed in the ^1^H NMR spectra (Supplementary Figure S13A). Imino proton region of the NOESY spectra exhibit the characteristic cross-peaks patterns of Watson–Crick GC or AT base pairs (Supplementary Figure S13C) and, most interestingly, H1’–H8 intraresidual NOEs for G2, A4 and G6 exhibit intensities comparable to those of cytosines H5–H6 NOEs (Supplementary Figure S13D), indicating that all glycosidic angles of purines are in syn. All these NMR data are fully consistent with the exclusive formation of the Z-form at 0 M NaCl ([oligo] = 1 mM) and demonstrate the ability of 2′-fluorinated nucleosides to act in synergy to overcome barriers to a B-to-Z transition in presence of TA interruptions. Future studies aim at exploring more TA interruption sites in the context of longer (CG)_*n*_ sequences.

### 2′-Fluorinated C/G duplexes bind to the Z_α_ domain of ADAR1

**Figure 9.**
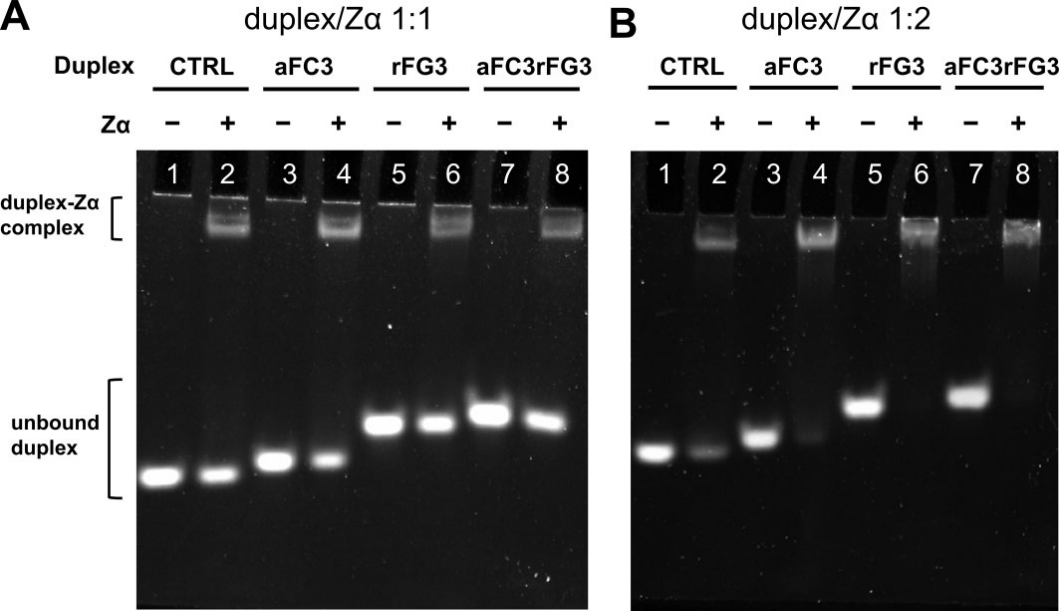
EMSA assays of **CTRL**, **aFC3**, **rFG3** and **aFC3rFG3** duplexes incubated with (**A**) equimolar amounts of Zα, and (**B**) 1:2 molar ratio of duplex to Zα (B). [Oligonucleotide] = 5 μM.

The crystal structure of the Zα domain of ADAR1p150 bound to Z-DNA shows primary contacts between the amino acid residues of Zα and the ‘zigzag’ phosphate backbone of Z-form duplex, indicating that this interaction is structure-specific (1). The increased propensity of 2′-fluorinated C/G duplexes to adopt the Z-form at lower salt concentrations encouraged us to study the relative binding affinities of **CTRL**, **aFC3**, **rFG3** and **aFC3rFG3** to the Zα domain using gel binding assays of duplex incubated with equimolar concentrations of Zα (Figure 9A) or 2-fold concentration of Zα (Figure 9B). The relative intensities of the gel bands corresponding to unmodified and modified duplexes with and without Zα indicate that fluorinated duplexes have higher relative binding affinities towards protein, in comparison to the native hexamer, consistent with their higher propensity to adopt Z-structure. Similar trends were also observed in the sequences containing an AT interruption (Table 2), whereby the intensity of bands corresponding to free nucleic acid was markedly reduced in the presence of Zα for the modified **FLUORO-TA** sequence, in comparison to unmodified **CTRL-TA** (Supplementary Figure S14A).

We also verified the negligible interference of the 2′-fluorine atoms in the protein binding properties of the sequences studied, using a non-Z forming sequence **G_3_C_3_** serving as the negative control, along with its 2′-fluorinated counterpart **FLUORO-G_3_C_3_** (Table 2). Both sequences exhibited minimal binding affinity towards protein, with only a small proportion of binding attributed to non-specific interactions between the protein and nucleic acid (Supplementary Figure S14B). Oligonucleotides with 2′F modifications have been utilized extensively in chemical biology and therapeutics, in probing interactions with numerous proteins including RISC, RNase H, thrombin, telomerase, and polymerases (75–84). Hence, the complexation of 2′F duplexes with Zα demonstrated in this study is yet another example of the biocompatibility of both araF and riboF nucleotide modifications.

In sum, a variety of spectroscopic and theoretical results show that the incorporation of the 2′-fluorinated nucleotides afC and rfG induces the formation of Z-form duplexes in (CG)_6_ sequences even in the absence of added salt. When these two substitutions are combined within the same strand, they synergistically facilitate a complete transition to the Z-form. The determination of the three-dimensional structures of these Z-form duplexes, in conjunction with molecular dynamics and quantum mechanics calculations, provides an explanation for the unprecedented stabilization effect caused by afC and rfG substitutions in the Z-form, as well as their synergistic impact. Notably, these sugar modifications are among the few that promote the B/Z transition and are compatible with conventional Z-form inducers featuring bulky C8 substitutions.

The fluorinated nature of the modified nucleosides allows for easy monitoring of B-Z transitions by ^19^F NMR spectroscopy. The distinct chemical shift differences enable the clear differentiation between B- and Z-forms and allows the reliable determination of their relative populations. This study opens the door to utilize ^19^F NMR to screen for novel Z-DNA binding proteins, thereby discovering whether other protein domains aside from Zα have specificity for Z-form duplexes and whether these are involved in regulating biological outcomes such as chromatin structure or RNA splicing (85). Moreover, stable Z-form duplexes under physiological conditions may find applications in the therapeutic space, such as improving the targeting of the ADAR1 editing enzyme.

## Supplementary Material

gkae508_Supplemental_File

## Data Availability

Coordinates are deposited in the PDB data bank under accession codes 8QDU (**aFC3**), 8QE4 (**rFG3**), and 8QOR (**aFC3rFG3**).
