# Supplementary material for: Formation of left-handed helices by C2′-fluorinated nucleic acids under physiological salt conditions
Source: Nucleic Acids Res. 2024 Jun 14;52(13):7414–28. doi: 10.1093/nar/gkae508 (PMC11260457; doi:10.1093/nar/gkae508)
Supplement: gkae508_Supplemental_File [file gkae508_supplemental_file.pdf]

Supplementary Data

Formation of left-handed helices by C2'-fluorinated nucleic acids under physiological salt conditions

AUTHORS

Roberto El-Khoury,[1,†] Cristina Cabrero,[2,†] Santiago Movilla,[3,‡] Harneesh Kaur,[1,‡] David Friedland,[1] Arnau Domínguez,[1,4] James D. Thorpe,[1] Morgane Roman,[1] Modesto Orozco,[3,*] Carlos González,[2,*] Masad J. Damha[1,*]

† Joint Authors
‡ Joint Authors

[1] Department of Chemistry, McGill University, Montreal, Quebec H3A 0B8, Canada
[2] Instituto de Química Física Blas Cabrera, CSIC, Serrano 119, 28006 Madrid, Spain
[3] Institute for Research in Biomedicine (IRB Barcelona), The Barcelona Institute of Science and Technology (BIST), 08028 Barcelona, Spain
[4] IQAC-CSIC, Jordi Girona 18-26, 08034 Barcelona, Spain

* To whom correspondence should be addressed. Email: masad.damha@mcgill.ca
Correspondence may also be addressed to cgonzalez@iqfr.csic.es and modesto.orozco@irbbarcelona.org.

Supplementary Figures

Figure S1. CD spectra of **aFC1**, **aFC2** and **aFC3** with increasing concentrations of NaCl. S-2
Figure S2. ^{19}F NMR spectra of **aFC1**, **aFC2** and **aFC3** with increasing concentrations of NaCl. S-3
Figure S3. ^{19}F-NMR spectra of **aFC3**, **rFG3** and the mix (**aFC3+rFG3**). S-4
Figure S4. Exchangeable proton region and NOESY spectra of **aFC3, rFG3** and **aFC3rFG3**. S-5
Figure S5. NOESY spectra of **aFC3 and rFG3**. S-6
Figure S6. DQF-COSY spectra of **aFC3, rFG3** and **aFC3rFG3**. S-7-8
Figure S7. Superposition and average of the structures calculated for **aFC3**, **rFG3** and **aFC3rFG3.** S-9
Figure S8. RMSD vs. simulation time for **aFC3**, **rFG3**, **aFC3rFG3** and **CTRL20**. S-10
Figure S9. RMSD vs. simulation time for **aFC10**, **rFG10**, **aFC10rFG10** and **CTRL20**. S-10
Figure S10. Average roll and twist angle along the MD simulations vs. base step. S-11
Figure S11. NMR spectra CGC(8-Br-rfG)CG (**rFBrG1**). S-12
Figure S12. CD spectra of FLUORO-TA (A) and CTRL-TA (B) with increasing concentrations of NaCl. S-13
Figure S13. NMR spectra of FLUORO-TA. S-14
Figure S14. EMSA assays of CTRL-TA, FLUORO-TA, CTRL, G_3C_3, and FLUORO-G_3C_3 with Zα. S-15

Supplementary Tables

Table S1. Chemical shifts list of **aFC3** (pH 7, T=15 °C, ^{19}F T=25 °C) S-16
Table S2. Chemical shifts list of **rFG3** (pH 7, T=5 °C, ^{19}F T=25 °C) S-16
Table S3. Chemical shifts list of **aFC3rFG3** (pH 7, T=5 °C, ^{19}F T=25 °C) S-16
Table S4. Experimental constraints and calculation statistics of **aFC3, rFG3** and **aFC3rFG3.** S-17
Table S5. Average dihedral angles and order parameters of the structure of **aFC3** at 0 M NaCl. S-17
Table S6. Average dihedral angles and order parameters of the structure of **rFG3** at 0 M NaCl. S-18
Table S7. Average dihedral angles and order parameters of the structure of **aFC3rFG3** at 0 M NaCl. S-18
Table S8. Intra and interresidual distances of **aFC3, rFG3** and **aFC3rFG3** at 0 M NaCl. S-18
Table S9. Characterization of bound critical points in the main interactions detected. S-19

Supplementary Methods

Synthesis and characterization of N^2-iBu-C8-bromo-2′-deoxy-2′-fluoro-guanosine (**2**) and its 3′-O-phosphoramidite derivative (**3**) S-20-27

Supplementary Figures

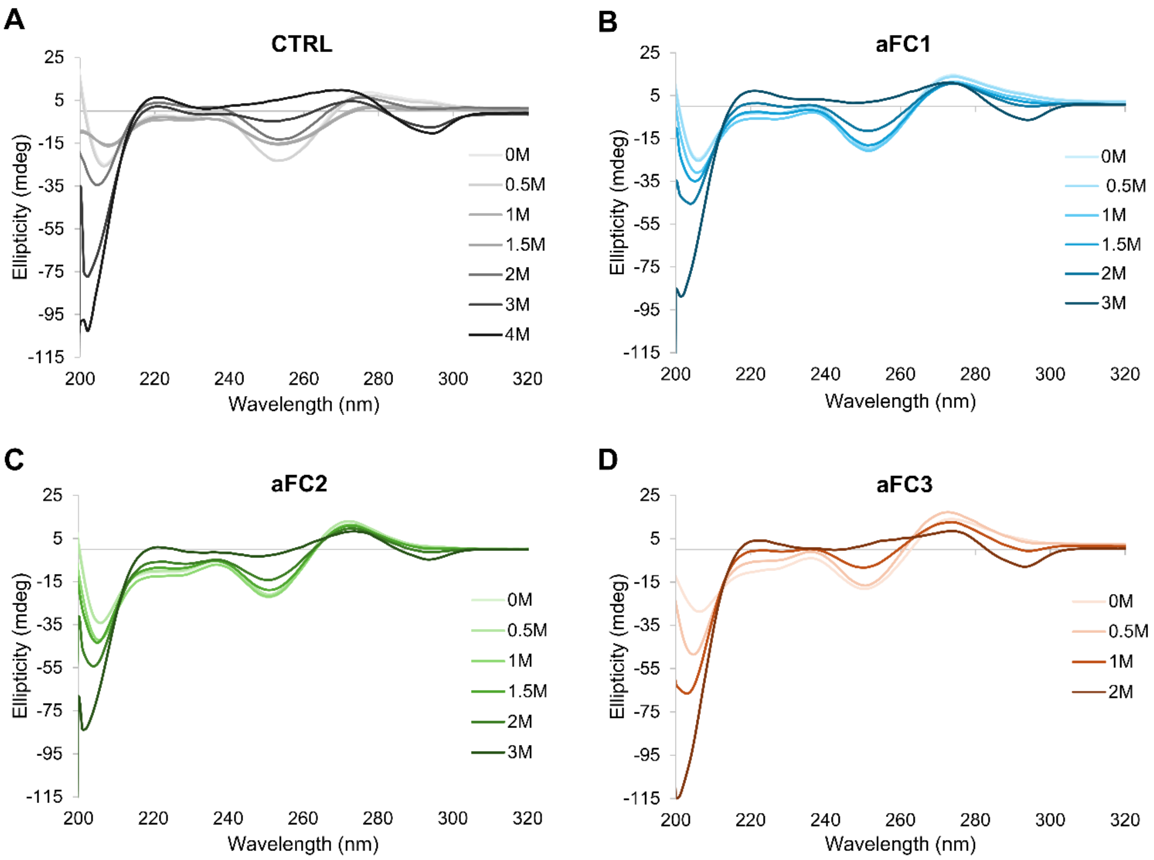

Figure S1. CD spectra superposition of **CTRL** (A), **aFC1** (B), **aFC2** (C) and **aFC3** (D) with increasing concentrations of NaCl. [oligonucleotide] = 125 μM. Experimental conditions: 10 mM sodium phosphate buffer, pH 7.0, T=25°C. Note: some precipitation is observed with **aFC2** and **aFC3** samples at 3M NaCl concentrations and the effect is increased at 4M NaCl concentrations.

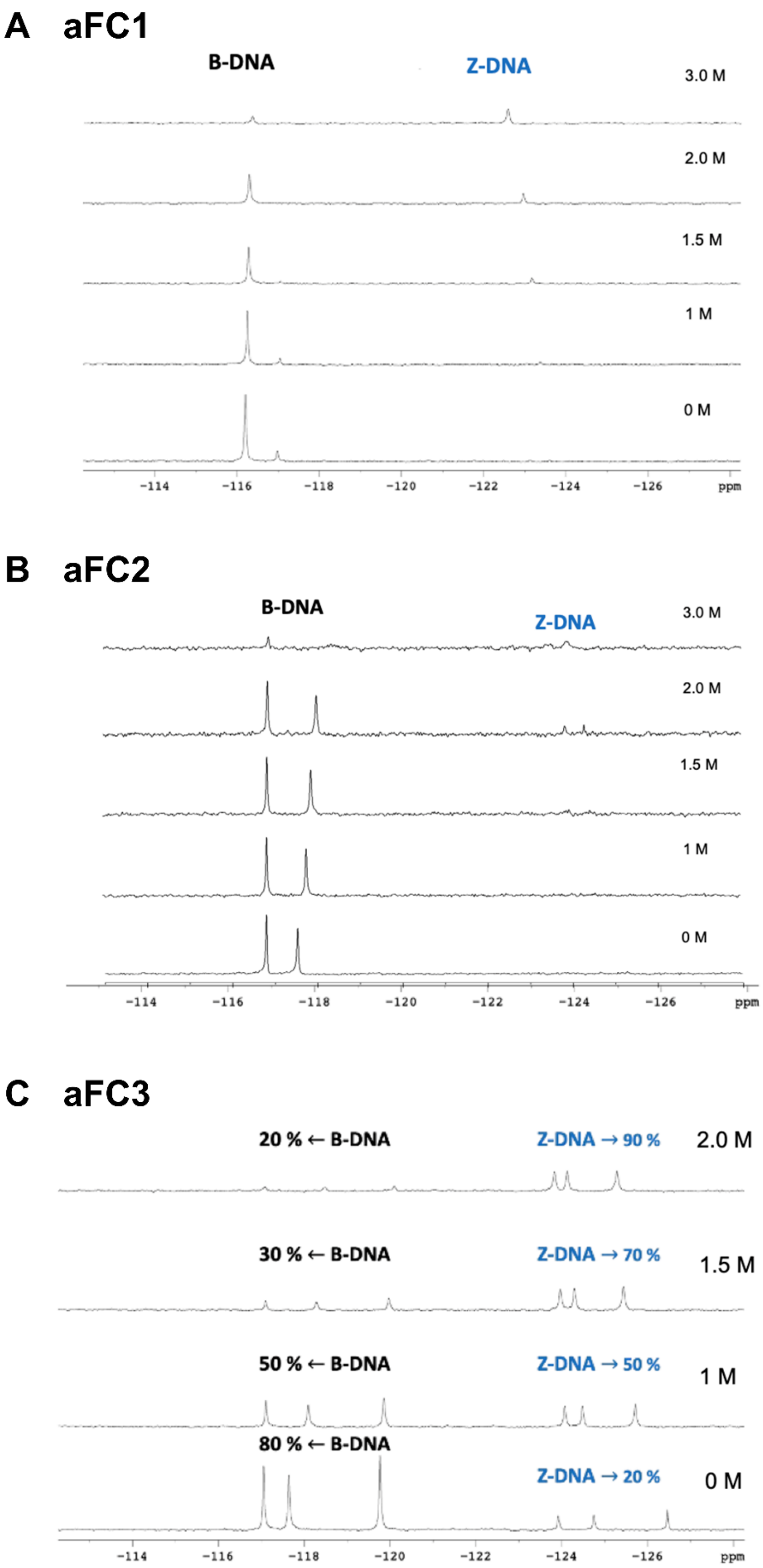

Figure S2. ^{19}F NMR spectra of **aFC1** (A), **aFC2** (B) and **aFC3** (C) with increasing concentrations of NaCl. Signals from B- and Z-forms can be distinguished by their salt dependence. The quality of the spectra significantly decreases at high salt concentrations (above 2 M), partly due to aggregation and partly due to the intrinsic sensitivity loss of NMR cryoprobes at high ionic strength. Despite this, the transitional midpoint for salt-induced transition could be estimated for **aFC3** to be 1 M, in complete agreement with CD data. Experimental conditions: 10 mM sodium phosphate buffer, pH 7.0, T=25°C.

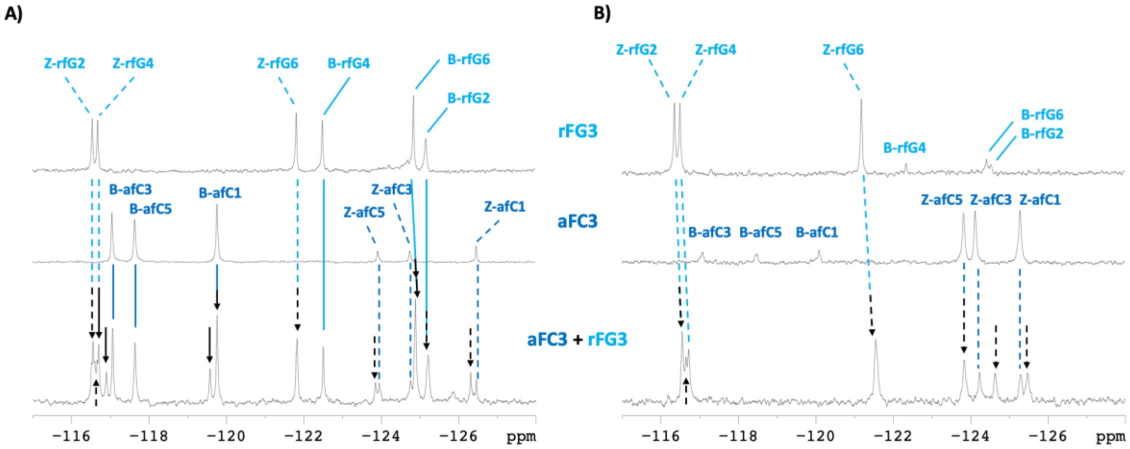

		aFC3	rFG3	aFC3:rFG3
0 M NaCl	B	22%	18%	22%
	Z	8%	19%	11%

		aFC3	rFG3	aFC3:rFG3
2 M NaCl	Z	14%	43%	43%

Figure S3. ^{19}F-NMR spectra of **aFC3**, **rFG3** and the mix (**aFC3+rFG3**) at T=25°C and 0 M (left) and 2 M NaCl (right). The signals of the homoduplex are shown in lines for the B-form and in dashed for the Z-form. The mix is shown with arrows. Experimental conditions: 10 mM sodium phosphate buffer, pH 7.0. [oligonucleotide] = 0.5 mM. The chemical shift assignments of the homoduplexes in the mixture are based on the spectra of the individual components alone (aFC3, rFG3: see Figures 2 and 3). The appearance of additional peaks is assigned to the **aFC3:rFG3** hybrid.

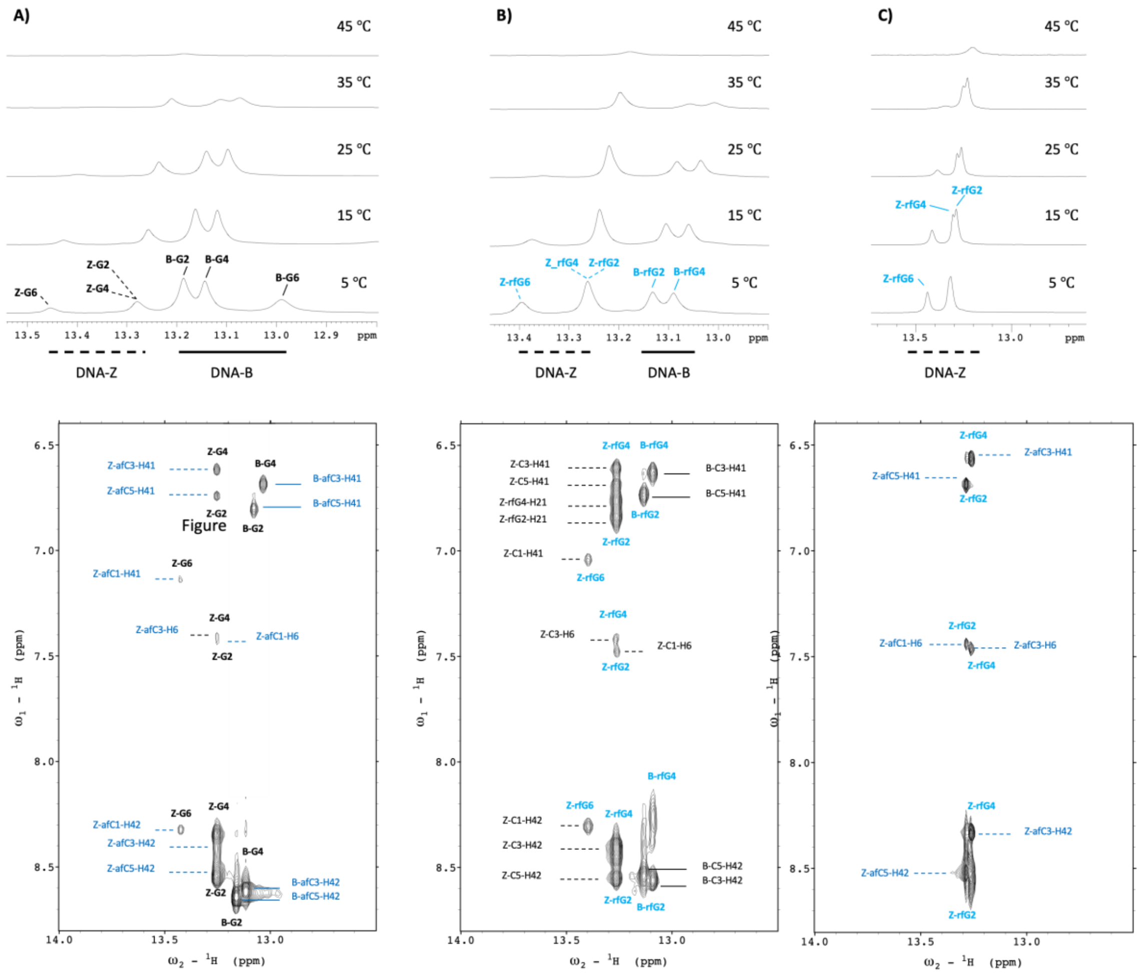

Figure S4.- ^{1}H-NMR spectra of **aFC3** (A), **rFG3** (B) and **aFC3rFG3** (C) at different temperatures, assigned from the NOESY spectra at T = 15℃, T = 5℃ and T =25℃, respectiively. Experimental conditions: 10 mM sodium phosphate buffer, pH 7.0. [oligonucleotide] = 0.5 mM.

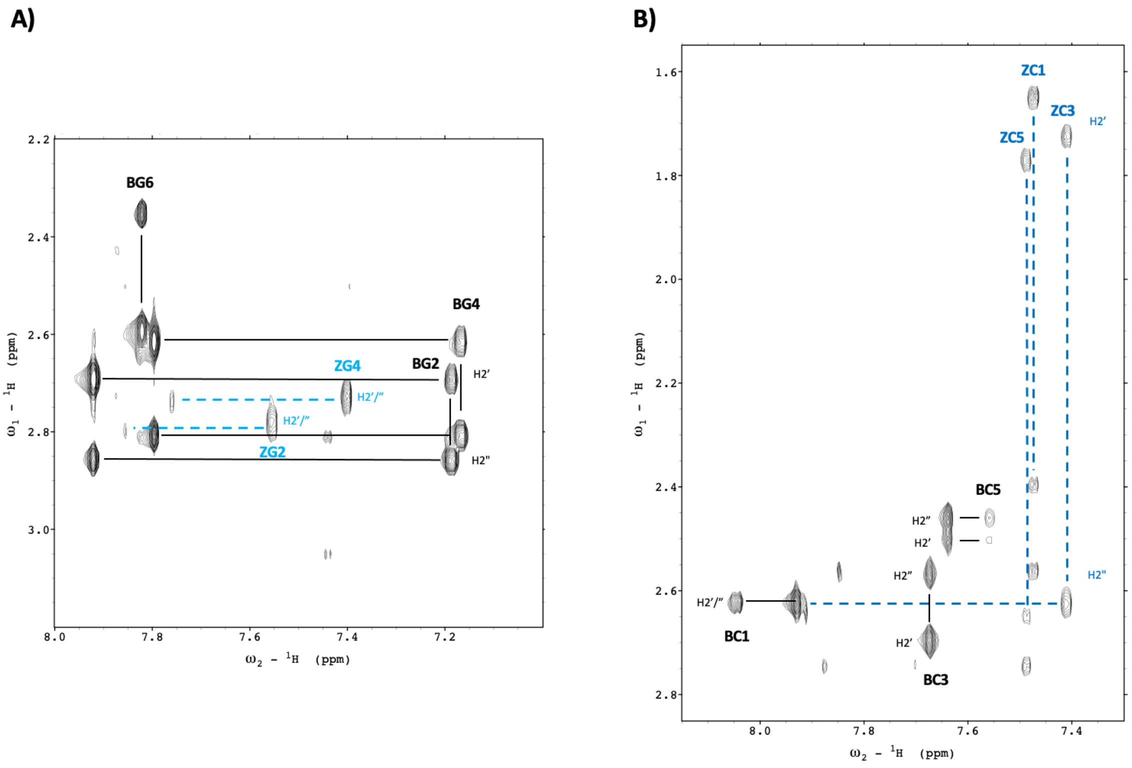

Figure S5. NOESY spectra of **aFC3** (A) and **rFG3** (B), indicating the sequential H2'/"-aromatic proton assignament. Experimental conditions: 10 mM sodium phosphate buffer, pH 7.0. [oligonucleotide] = 0.5 mM.

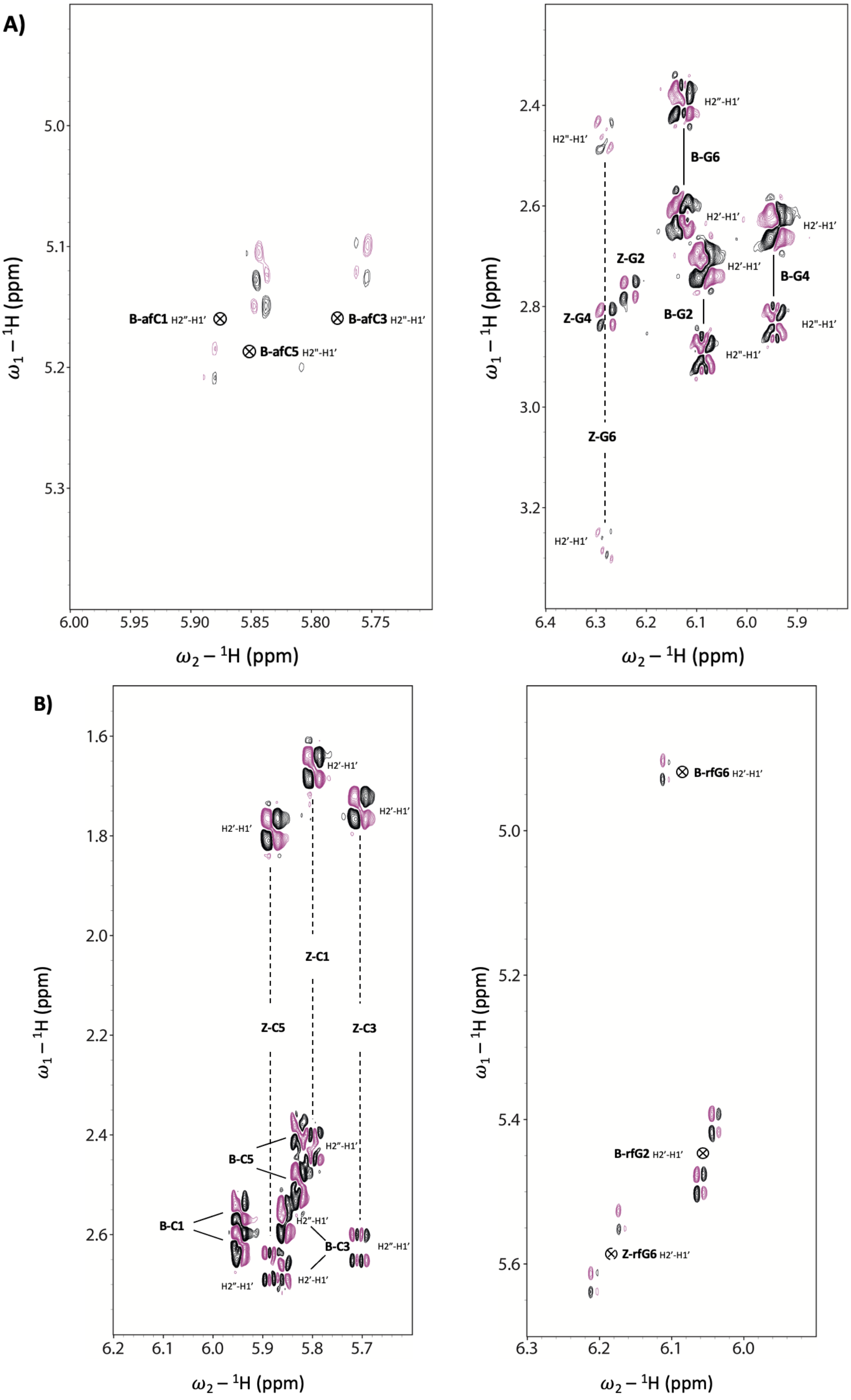
A)
B-afC1 H2″-H1′
B-afC5 H2″-H1′
B-afC3 H2″-H1′
B-G6
B-G4
B-G2
Z-G2
Z-G4
Z-G6
B)
Z-C1
Z-C5
Z-C3
B-C5
B-C1
B-C3
B-rfG6 H2′-H1′
B-rfG2 H2′-H1′
Z-rfG6 H2′-H1′
$\omega_1 - {}^1H$ (ppm)
$\omega_2 - {}^1H$ (ppm)

C)

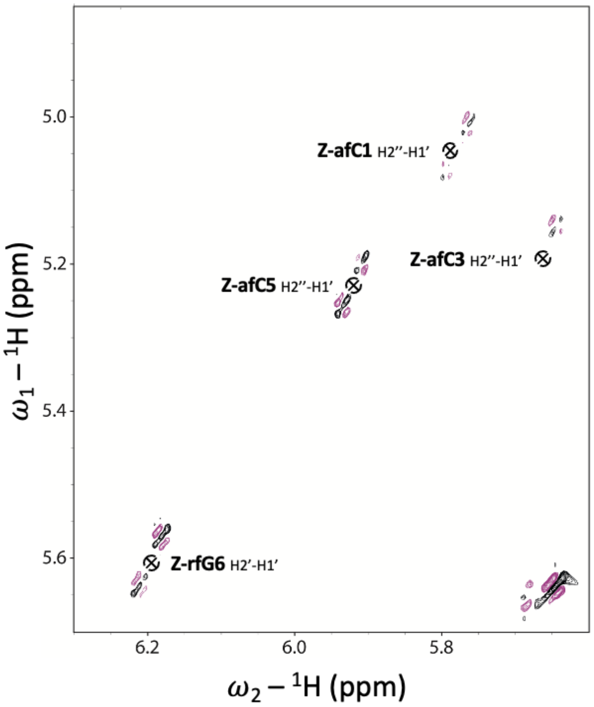

Figure S6.- DQF-COSY spectra of **aFC3** (A), **rFG3** (B) and **aFC3rFG3** (C), showing the assigments of the cytosines (left) and the guanines (right). Experimental conditions: 10 mM sodium phosphate buffer, pH 7.0, T=25°C. [oligonucleotide] = 0.5 mM.

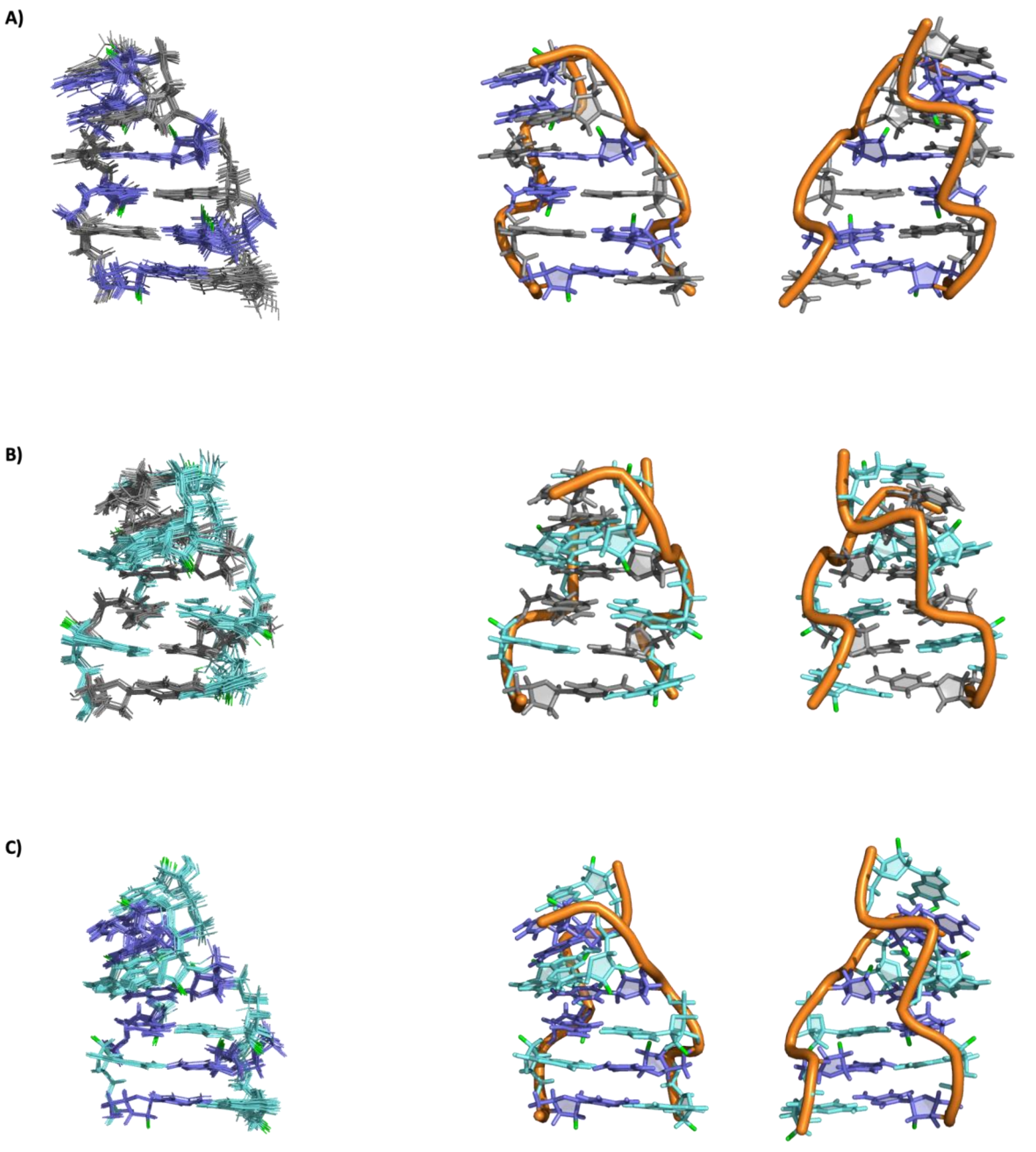

Figure S7.- Superposition and average structures of the Z-forms of A) **aFC3**, B) **rFG3**, and C) **aFC3rFG3**. Experimental conditions: 10 mM sodium phosphate buffer, pH 7.0. [oligonucleotide] = 0.5 mM.

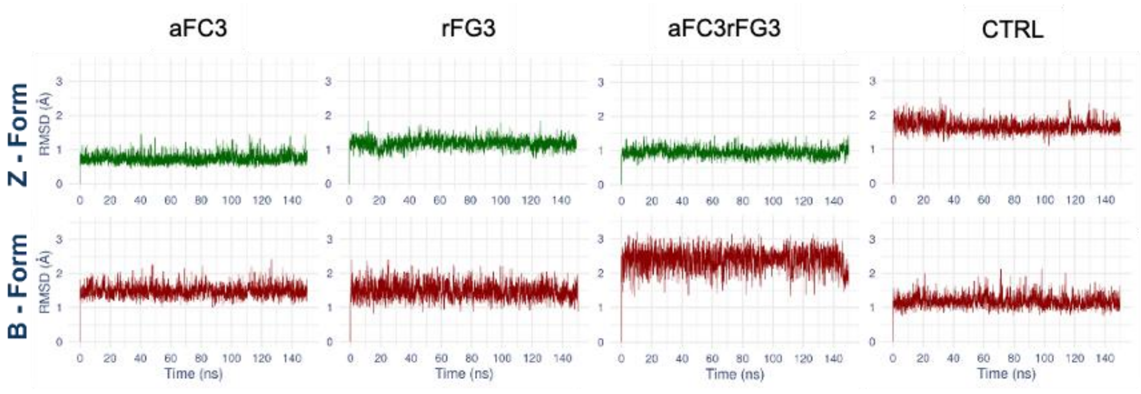

Figure S8. Root-mean-square deviation (RMSD) in Angstroms vs. simulation time (ns) for the oligonucleotides **aFC3**, **rFG3**, **aFC3rFG3** and **CTRL** starting from B and Z conformations. With green lines are presented those simulations that started from NMR experimental structures and with red those which started from generated models.

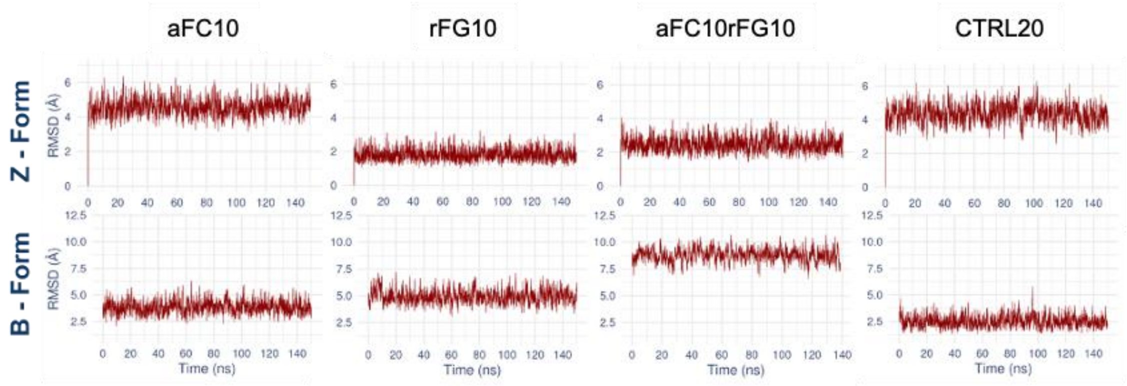

Figure S9. Root-mean-square deviation (RMSD) in Angstroms vs. simulation time (ns) for the **aFC10**, **rFG10**, **aFC10rFG10** and **CTRL20** strands starting from B and Z canonical conformations. All these simulations started from generated models.

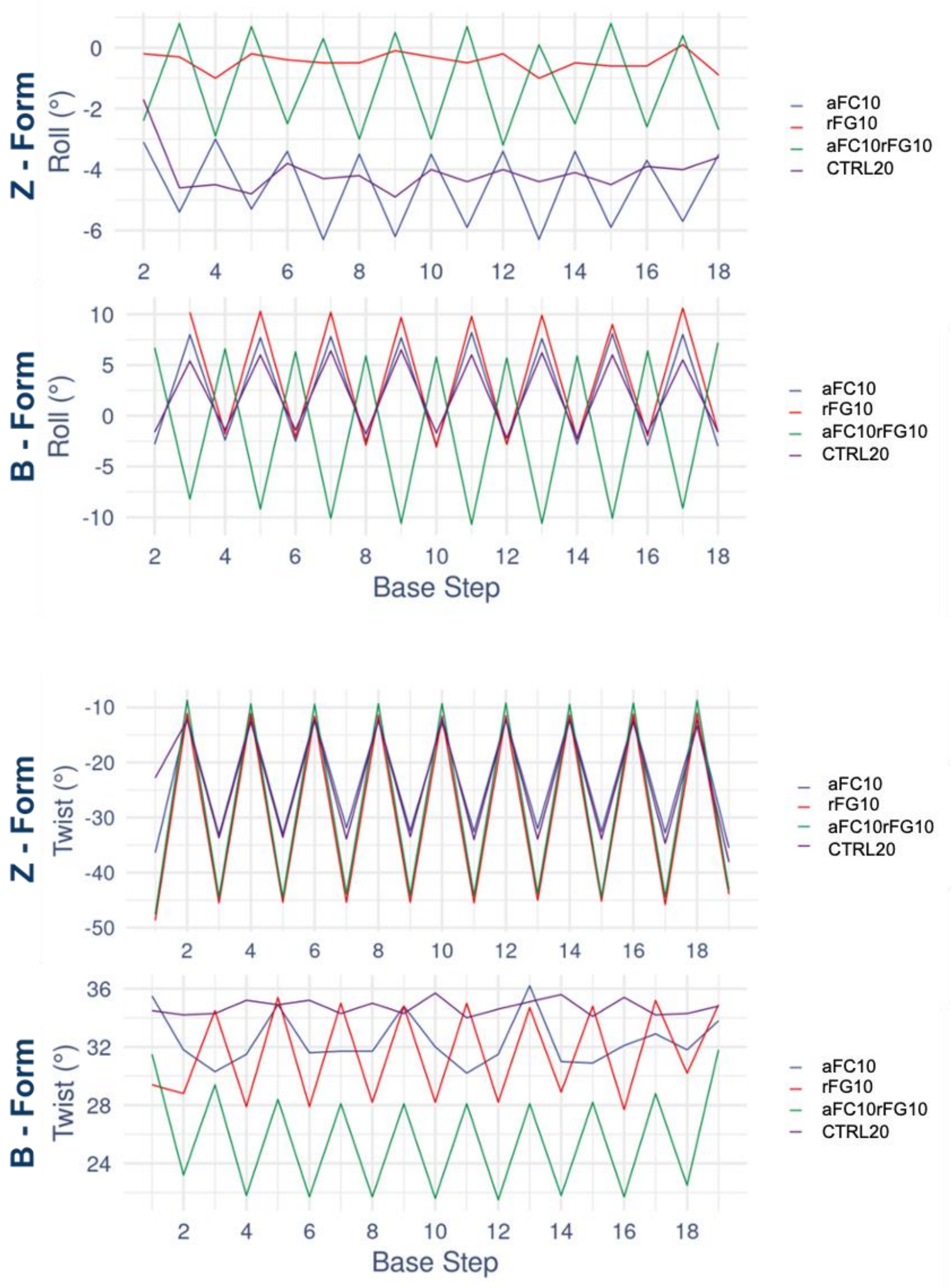

Figure S10. Average roll (first two rows) and twist (last two rows) angle along the MD simulations vs. base step for the aFC10, rFG10, aFC10-rFG10 and CTRL20 strands starting from B and Z canonical conformations.

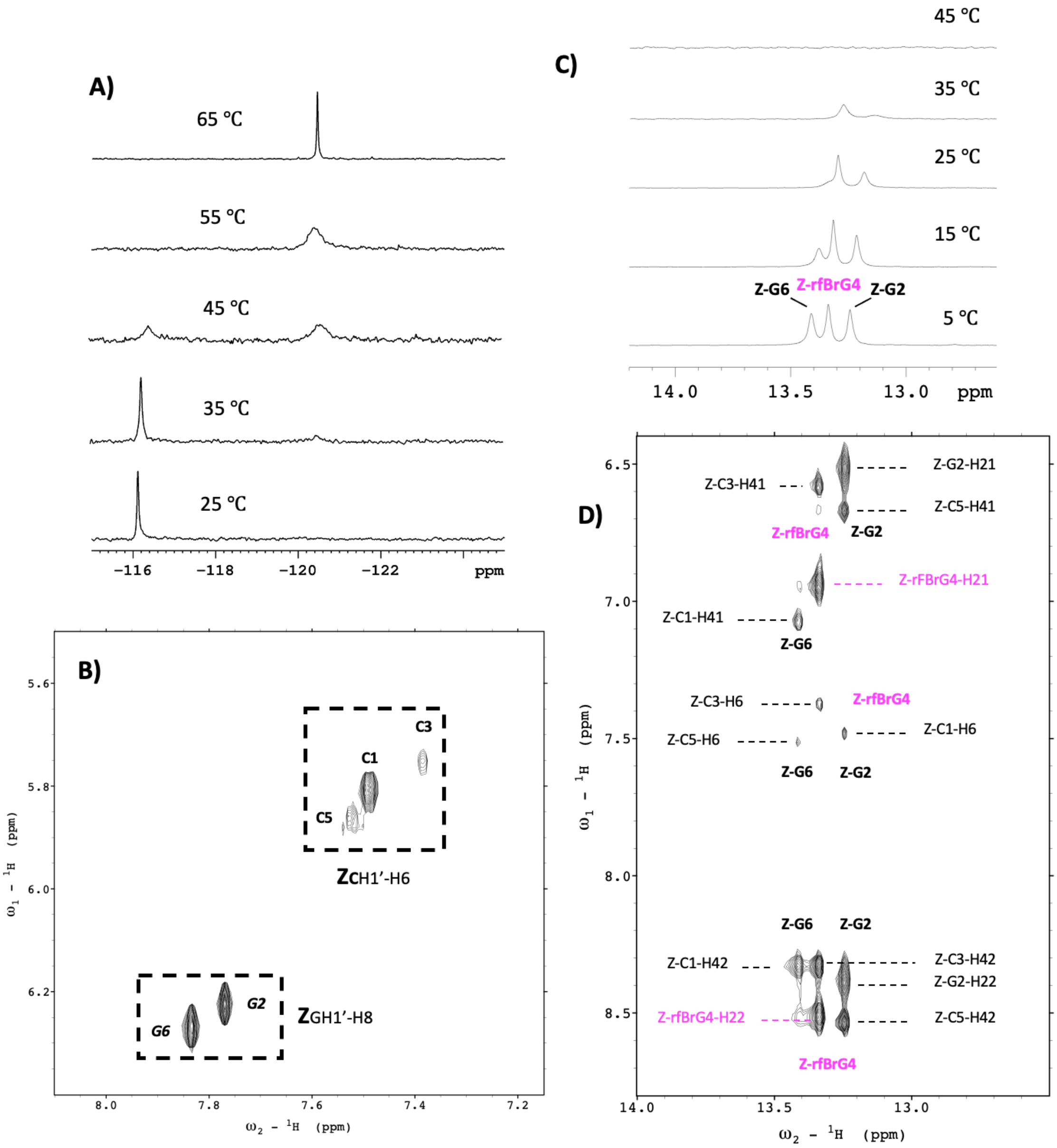

Figure S11. A) ^{19}F-NMR spectra of CGC(8-Br-rfG)CG (**rFBrG1**) at different temperatures. ^{19}F signal at -116 ppm is in the region corresponding to riboF-G in the Z-form (see Figure 3B in the main text). The signal at -120.5 ppm corresponds to the unfolded oligonucleotide. B) H1'/H5 – H6/H8 region of the NOESY spectra. The strong H1'/H8 NOEs indicate that the guanines are in *syn* conformation. C) Imino proton region of the 1H-NMR spectra at different temperatures. D) Exchangeable proton region of the NOESY spectra (T = 5C). Cross-peak patterns are totally consistent with a single species. Experimental conditions: 10 mM sodium phosphate buffer, pH 7.0. [oligonucleotide] = 0.5 mM.

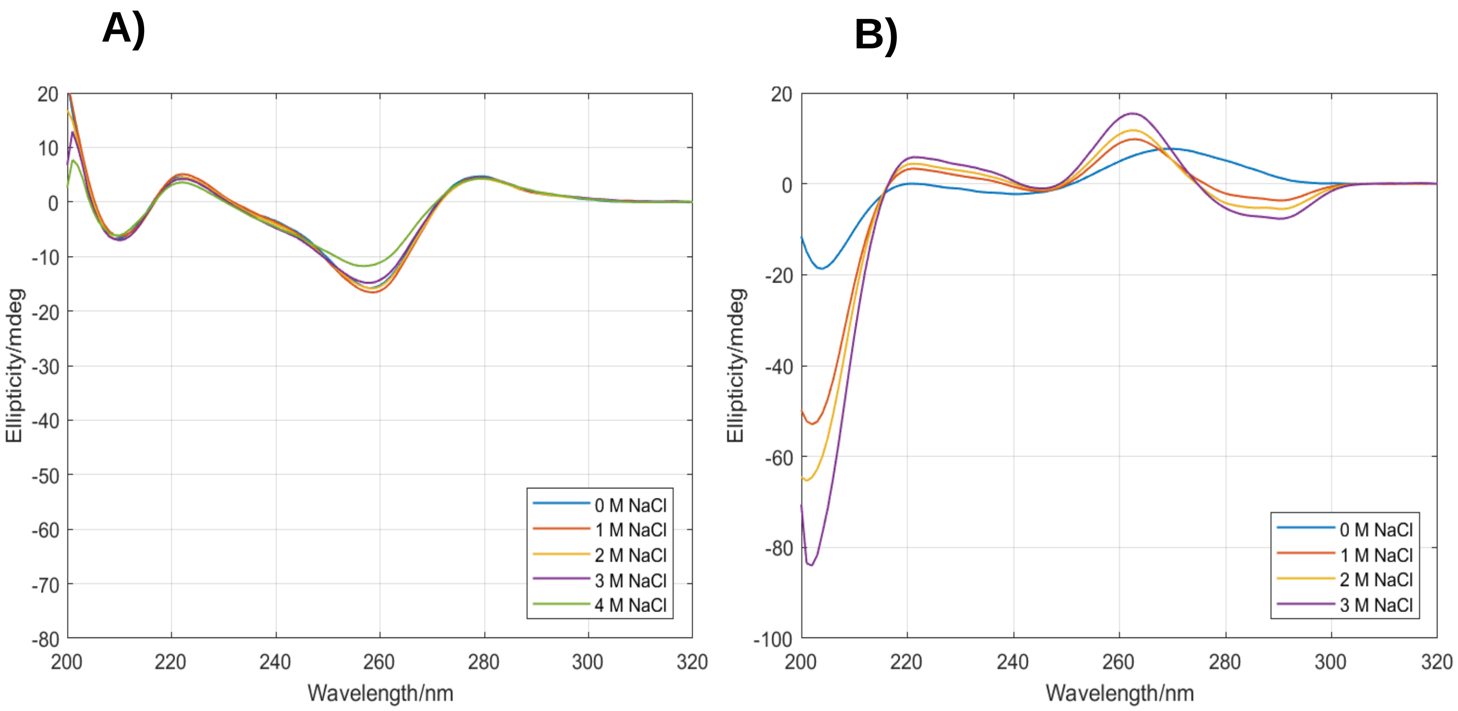

Figure S12. CD spectra of **CTRL-TA** (A) and **FLUORO-TA** (B) with increasing concentrations of NaCl. [oligonucleotide] = 125 μM.

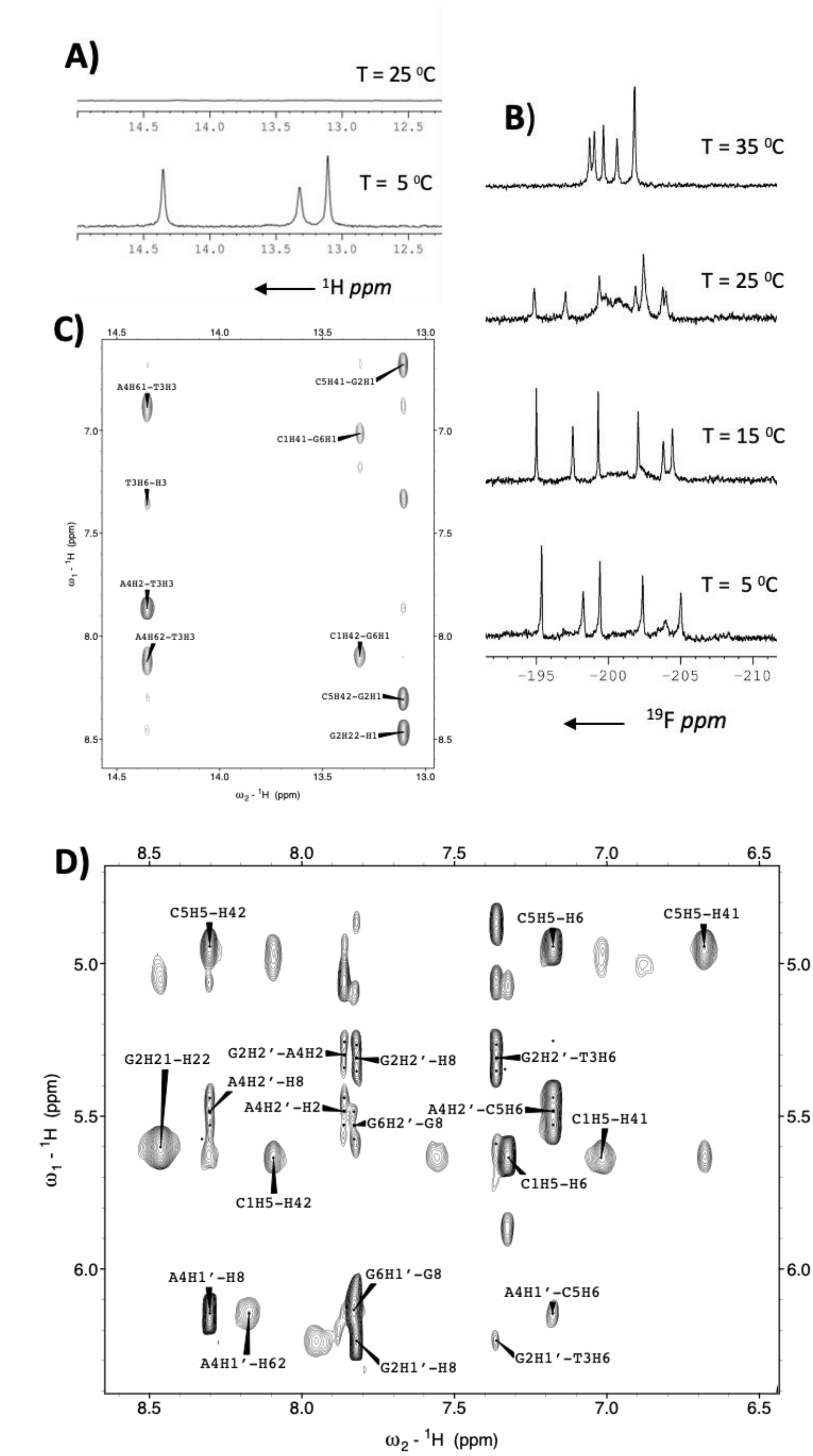

Figure S13. (A) ^{1}H-NMR spectra of **FLUORO-TA** at 5 °C and 25 °C. (B) ^{19}F-NMR spectra at different temperatures. (C) Imino proton region of the NOESY spectra showing characteristic NOE pattern of Watson-Crick GC or AT base pairs (τm=150 ms , T = 5 ºC). (D) H1'-aromatic region of the NOESY spectra of **FLUORO-TA**. H1'-H8 intraresidual cross-peaks for G2, A4 and G6 exhibit a high intensity, comparable to cytosines H5-H6 NOEs, indicating that the glycosidic angles are in syn. [oligonucleotide] = 1 mM. Buffer conditions: 10 mM NaPi, pH 7.0.

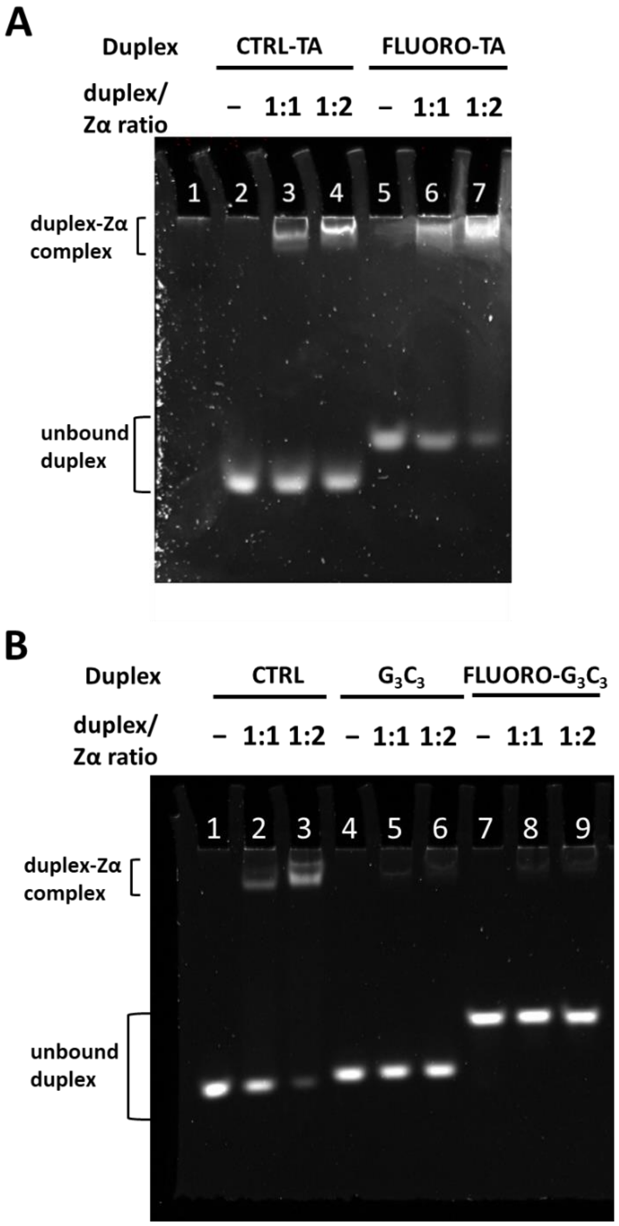

Figure S14. EMSA assays of **CTRL-TA** and **FLUORO-TA** (A) and **CTRL**, **G_3C_3**, and **FLUORO-G_3C_3** (B) incubated alone, with equimolar amounts of Zα, or 1:2 molar rations of duplex to Zα. [oligonucleotide] = 5 μM. In (A), lane 1 contains a mixture of xylene cyanol and bromothymol blue for tracking the progress of electrophoresis.

Supplementary Tables

Table S1.- Chemical shifts of **aFC3**, pH 7, T=15 °C, ^{19}F T=25 °C

B-form	H1	H42/H22	H41/H21	H6/H8	H5	H1’	H2’	H2”	F	H3’	H4'	H5'	H5"
afC1/afC7	-	7.91	7.03	7.61	5.89	5.86	-	5.14	-119.8	4.65	4.06	3.80	3.89
G2/G8	13.16	8.69	6.66	7.92	-	6.08	2.70	2.86	-	5.00	4.40	4.05	4.15
afC3/afC9	-	8.61	6.60	7.19	5.28	5.77	-	5.12	-117.0	4.83	4.08	4.16	4.43
G4/G10	13.12	8.60	6.72	7.80	-	5.95	2.62	2.81	-	5.01	4.37	4.03	4.17
afC5/afC11	-	8.64	6.72	7.17	5.31	5.84	-	5.15	-117.6	4.75	4.13	4.12	4.42
G6/G12	12.81	n. o.	n. o.	7.82	-	6.12	2.59	2.35	-	4.67	4.13	4.11	4.12

Z-form	H1	H42/H22	H41/H21	H6/H8	H5	H1’	H2’	H2”	F	H3’	H4'	H5'	H5"
afC1/afC7	-	8.33	7.14	7.44	5.83	5.78	-	5.05	-126.5	4.59	3.72	2.81	3.05
G2/G8	13.25	8.50	6.85	7.76	-	6.24	2.74	2.74	-	4.88	4.23	4.09	4.21
afC3/afC9	-	8.35	6.61	7.40	5.08	5.69	-	5.18	-124.7	4.91	3.84	2.50	3.71
G4/G10	13.25	8.53	6.48	7.86	-	6.28	2.79	2.79	-	4.89	4.24	4.14	4.22
afC5/afC11	-	8.55	6.74	7.56	5.24	5.92	-	5.22	-123.9	4.96	3.93	2.73	3.78
G6/G12	13.43	n. o.	n. o.	7.87	-	6.28	3.25	2.43	-	4.80	4.28	4.15	4.20

n.o.: not observed

Table S2.- Chemical shifts of **rFG3**, pH 7, T=5 °C, ^{19}F T=25 °C

B-form	H1	H42/H22	H41/H21	H6/H8	H5	H1’	H2’	H2”	F	H3’	H4'	H5'	H5"
C1/C7	-	8.21	7.11	8.05	6.04	5.89	2.62	2.62	-	4.73	4.16	3.92	3.98
rfG2/rfG8	13.13	8.46	6.65	7.93	-	6.07	5.41	-	-125.2	4.92	4.56	4.21	4.38
C3/C9	-	8.56	6.63	7.66	5.37	5.83	2.70	2.57	-	4.71	4.26	4.22	4.39
rfG4/rfG10	13.09	8.40	6.68	7.67	-	6.01	5.24	-	-122.5	4.77	4.50	4.16	4.38
C5/C11	-	8.53	6.73	7.56	5.33	5.80	2.50	2.50	-	4.67	4.19	4.12	4.34
rfG6/rfG12	n. o.	n. o.	n. o.	7.64	-	6.13	4.93	-	-124.9	4.51	4.25	4.09	4.28

Z-form	H1	H42/H22	H41/H21	H6/H8	H5	H1’	H2’	H2”	F	H3’	H4'	H5'	H5"
C1/C7	-	8.30	7.04	7.47	5.81	5.80	1.65	2.40	-	4.58	3.74	2.56	3.13
rfG2/rfG8	13.26	8.41	6.84	7.85	-	6.22	5.29	-	-116.6	5.25	4.33	4.11	4.17
C3/C9	-	8.39	6.61	7.41	5.05	5.73	1.73	2.61	-	4.85	3.88	2.63	3.89
rfG4/rfG10	13.26	8.45	6.76	7.91	-	6.25	5.31	-	-116.7	5.23	4.33	4.16	4.22
C5/C11	-	8.55	6.68	7.49	5.17	5.88	1.77	2.65	-	4.86	4.01	2.75	3.91
rfG6/rfG12	13.4	n. o.	n. o.	7.88	-	6.19	5.54	-	-121.8	5.21	4.27	4.20	4.25

n.o.: not observed

Table S3.- Chemical shifts of **aFC3rFG3**, pH 7, T=5 °C, ^{19}F T=25 °C

Z-form	H1	H42/H22	H41/H21	H6/H8	H5	H1’	H2’	H2”	F	H3’	H4'	H5'	H5"
afC1/afC7	-	8.28	7.09	7.44	5.84	5.77	-	5.05	-126.3	4.57	3.81	2.84	3.08
rfG2/rfG8	13.28	8.42	6.09	7.84	-	6.18	5.24	-	-116.1	4.88	4.30	4.12	4.18
afC3/afC9	-	8.33	6.57	7.47	5.10	5.66	-	5.19	-125.2	4.89	3.96	2.58	3.76
rfG4/rfG10	13.26	8.55	5.97	7.93	-	6.26	5.33	-	-116.1	4.85	4.32	4.15	4.23
afC5/afC11	-	8.52	6.69	7.70	5.26	5.91	-	5.23	-124.2	4.96	4.06	2.80	3.77
rfG6/rfG12	13.39	8.55	6.00	7.92	-	6.19	5.61	-	-121.8	5.14	4.25	2.82	3.81

n.o.: not observed

Table S4.- Experimental constraints and calculation statistics of **aFC3**, **rFG3** and **aFC3rFG3** at 0 M NaCl.

**Z-Form**	**aFC3**		**rFG3**		**aFC3rFG3**	
**Experimental distance constraints**						
Total number	75		74		111	
intra-residue	25		30		29	
sequential	28		36		48	
range > 1	22		8		34	
**RMSD (Å)**						
all well-defined* bases	0.29 ± 0.11		0.25 ± 0.08		0.17 ± 0.06	
all well-defined* heavy atoms	0.49 ± 0.18		0.51 ± 0.18		0.39 ± 0.13	
backbone	0.8 ± 0.4		0.61 ± 0.17		0.47 ± 0.17	
all heavy atoms	0.7 ± 0.3		0.54 ± 0.16		0.43 ± 0.14	
Residual violations	Average	Range	Average	Range	Average	Range
Sum of violation (Å)	2.06	1.82…2.41	3.26	2.92…3.84	Average	Range
Max. violation (Å)	0.39	0.35…0.44	0.20	0.19…0.23	6.80	6.56…7.41
NOE energy# (kcal/mol)	11.44	10.22…14.49	10.07	8.16…12.79	0.45	0.43…0.46
Total energy (kcal/mol)	-741	-971…-593	-713	-917…-643	41.63	40.28…45.26
K_{NOE} = 100 kcal/(mol·Å^2)						

Table S5.- Average dihedral angles and order parameters of the structure of **aFC3** at 0 M NaCl.

**Z-form**	**Pseudorot.**		**α**		**β**		Y		**δ**		**ε**		**ζ**		**X**	
	Phase	Ampl.	Ave.	OP	Ave.	OP	Ave.	OP	Ave.	OP	Ave.	OP	Ave.	OP	Ave.	OP
afC1	124	41	-	-	-	-	58	1.0	120	1.0	-86	1.0	-	-	-172	1.0
G2	131	21	76	1.0	-177	1.0	-176	1.0	123	1.0	-140	1.0	69	1.0	61	1.0
afC3	121	33	-169	1.0	-165	1.0	61	1.0	118	1.0	-85	1.0	-40	1.0	-172	1.0
G4	127	27	73	1.0	-174	1.0	-179	1.0	121	1.0	-135	1.0	70	1.0	70	1.0
afC5	125	32	-166	1.0	-168	1.0	62	1.0	121	1.0	-87	1.0	-44	1.0	-171	1.0
G6	162	40	77	1.0	-177	1.0	-174	1.0	148	1.0	-	-	67	1.0	78	1.0
afC7	125	41	-	-	-	-	58	1.0	121	1.0	-87	1.0	-	-	-172	1.0
G8	131	20	76	1.0	-177	1.0	-175	1.0	121	1.0	-138	1.0	69	1.0	61	1.0
afC9	120	33	-172	1.0	-154	1.0	62	1.0	117	1.0	-84	1.0	-46	1.0	-172	1.0
G10	133	27	73	1.0	-175	1.0	-178	1.0	124	1.0	-137	1.0	69	1.0	71	1.0
afC11	122	31	-171	1.0	-165	1.0	63	1.0	119	1.0	-86	1.0	-45	1.0	-170	1.0
G12	161	39	76	1.0	-178	1.0	-175	1.0	147	1.0	-	-	68	1.0	80	1.0

Table S6.- Average dihedral angles and order parameters of the structure of **rFG3** at 0 M NaCl.

**Z-form**	**Pseudorot.**		**α**		**β**		Υ		**δ**		**ε**		**ζ**		**Χ**	
	Phase	Ampl.	Ave.	OP	Ave.	OP	Ave.	OP	Ave.	OP	Ave.	OP	Ave.	OP	Ave.	OP
C1	142	50					50	0.8	140	1.0	-90	1.0			-161	1.0
rfG2	23	21	73	1.0	-176	1.0	174	1.0	93	1.0	-132	1.0	70	1.0	62	1.0
C3	138	49	-152	1.0	-136	1.0	56	1.0	135	1.0	-88	1.0	-51	0.9	-155	1.0
rfG4	28	21	68	1.0	-178	1.0	175	1.0	94	1.0	-113	0.9	75	1.0	63	1.0
C5	179	43	-23	1.0	178	0.8	-65	1.0	159	1.0	-64	1.0	-54	0.6	-159	1.0
rfG6	145	46	-128	1.0	162	1.0	39	1.0	135	1.0			76	1.0	-60	1.0
C7	145	49					36	0.7	142	1.0	-90	1.0			-157	1.0
rfG8	29	21	73	1.0	-174	1.0	176	1.0	93	1.0	-137	1.0	69	1.0	62	1.0
C9	138	49	-157	1.0	-135	1.0	58	1.0	135	1.0	-88	1.0	-47	1.0	-156	1.0
rfG10	33	21	71	1.0	-179	1.0	174	1.0	92	1.0	-115	0.9	72	1.0	62	1.0
C11	176	43	-11	0.5	172	0.8	-66	1.0	158	1.0	-75	1.0	-58	0.6	-164	1.0
rfG12	157	46	81	0.5	-167	0.9	155	0.5	144	1.0			70	1.0	-67	1.0

Table S7.- Average dihedral angles and order parameters of the structure of **aFC3rFG3** at 0 M NaCl.

**Z-form**	**Pseudorot.**		**α**		**β**		Υ		**δ**		**ε**		**ζ**		**Χ**	
	Phase	Ampl.	Ave.	OP	Ave.	OP	Ave.	OP	Ave.	OP	Ave.	OP	Ave.	OP	Ave.	OP
afC1	118	42					59	1.0	114	1.0	-78	1.0			-175	1.0
rfG2	25	17	68	1.0	177	1.0	170	1.0	99	1.0	-102	1.0	76	1.0	65	1.0
afC3	135	35	-87	1.0	172	1.0	46	1.0	125	1.0	-86	1.0	-66	1.0	-176	1.0
rfG4	34	19	73	1.0	-180	1.0	179	1.0	97	1.0	-111	0.7	73	1.0	51	1.0
afC5	129	41	-134	0.8	159	1.0	50	1.0	123	1.0	-84	1.0	-17	0.6	-155	1.0
rfG6	51	25	72	0.9	-174	1.0	170	0.9	92	1.0			74	1.0	67	1.0
afC7	117	42					60	1.0	114	1.0	-79	1.0			-175	1.0
rfG8	23	17	68	1.0	177	1.0	170	1.0	99	1.0	-100	1.0	77	1.0	65	1.0
afC9	135	36	-89	1.0	171	1.0	48	1.0	126	1.0	-79	1.0	-66	1.0	-176	1.0
rfG10	55	34	-147	0.9	173	1.0	55	0.9	83	1.0	-133	1.0	77	1.0	64	1.0
afC11	130	39	-142	0.9	-150	0.9	56	1.0	124	1.0	-85	1.0	-54	1.0	-158	1.0
rfG12	49	23	68	1.0	-174	1.0	174	1.0	94	1.0			74	1.0	68	1.0

Table S8.- Average values of relevant contacts in the Z-form structures of **aFC3**, **rFG3** and **aFC3rFG3**.

INTRARRESIDUAL N3··H3'				INTERRESIDUAL H22··F			
RESIDUES	**aFC3**	**rFG3**	**aFC3rFG3**	RESIDUES	**aFC3**	**rFG3**	**aFC3rFG3**
2 G	3.9	2.5	2.7	2 G -3 C	2.8	-	2.2
4 G	4.0	2.6	2.9	4 G -5 C	2.8	-	1.9
6 G	4.7	4.9	3.0				
8 G	3.8	2.6	2.7	8 G -9 C	2.8	-	2.2
10 G	4.1	2.6	3.0	10 G -11 C	2.8	-	1.9
12 G	4.6	5.0	2.9				

Table S9.- Averages of electron densities ρ(**r**), potential energy densities V(**r**), total energy densities E(**r**) and reduced density gradients of the bound critical points (BCP) associated with the main interactions detected in the reduced models aF-rF, aF and rF. All values are reported in atomic units.

Model	Connected atoms	*ρ(r)*	*V(r)*	*E(r)*	RDG
aF-rF	(F2'|aF-C)-(H21|rF-G)	2.04E-02	-1.69E-02	1.99E-03	3.54E-15
	(H3'|rF-G)-(N3|rF-G)	7.12E-03	-3.75E-03	8.64E-04	2.53E-15
aF	(F2'|aF-C)-(H21|rF-G)	2.60E-03	-1.61E-03	7.43E-04	9.08E-15
rF	(H3'|rF-G)-(N3|rF-G)	7.66E-03	-4.06E-03	9.26E-04	5.10E-15

SUPPLEMENTARY METHODS

Synthesis of N^2-iBu-C8-bromo-2'-fluoro-guanosine 3'-O-phosphoramidite (compound **3**).

Step 1:

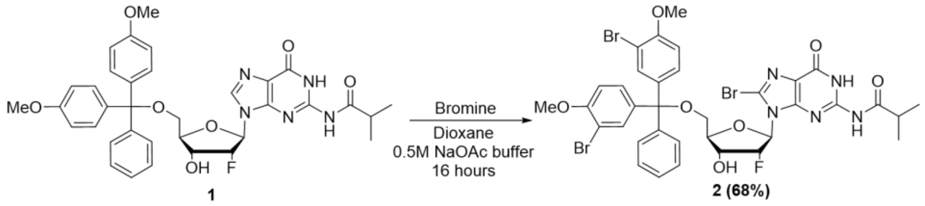

Step 2:

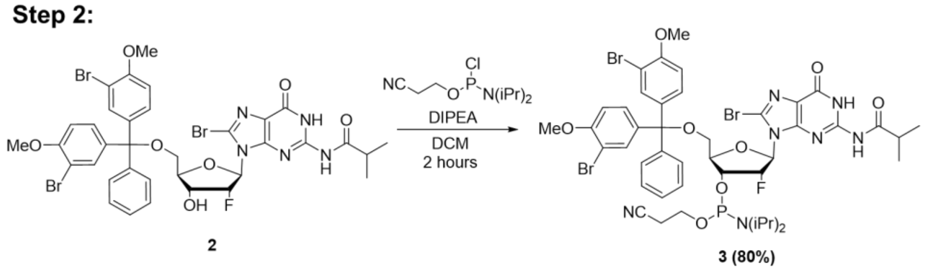

Compound **1** (0.5 g, 0.75 mmol) was dissolved in 20 mL of dry dioxane and 15 mL of a pH 5.4, 0.5M sodium acetate buffer. While stirring at room temperature, bromine (0.08 mL, 1.5 mmol) was added in four equal portions (0.02 mL each) every 15 minutes. The reaction was allowed to stir overnight for 16 hours. After reaction completion was verified by TLC (consumption of starting material and formation of a faster migrating spot, 5% MeOH/DCM), the reaction was quenched with a 20 mL solution of 10% aqueous sodium thiosulfate then washed with 30 mL of saturated aqueous sodium bicarbonate. The aqueous layers were back extracted with DCM and the organic layers were combined, dried over sodium sulfate and concentrated. The residue was dissolved in a small amount of DCM and purified by column chromatography (gradient, 0-3% MeOH/DCM). The appropriate fractions were combined and concentrated to yield compound **2** as an off-white solid (0.53g, 68%).

HRMS calc. [M+Na] 913.9806. HRMS found ESI+ [M+Na]+ 913.9792. The ^{1}H NMR and ^{13}C NMR spectra are displayed in **Figures S15 and S16**, respectively. ^{1}H-NMR δH (500 MHz, $CDCl_3$) 0.74 (d, 3H, J = 6.8 Hz), 0.99 (d, 3H, J = 6.6 Hz), 1.09 (t, 12H, J = 7.3 Hz), 3.18 (dd, 1H, J = 11.0, 3.9 Hz), 3.55 (dd, 1H, J = 10.9, 1.8 Hz), 3.87 (s, 3H), 3.88 (s, 3H), 6.06 (dt, 1H, J = 54.0, 4.5 Hz), 6.19 (dd, 1H, J = 16.7, 4.2 Hz), 6.80 (dd, 2H, J = 8.9, 4.5 Hz), 7.26 (m, 1H), 7.30 (m, 3H), 7.43 (m, 2H), 7.65 (d, 1H, J = 2.4 Hz), 7.73 (d, 1H, J = 2.2 Hz). ^{13}C-NMR δC (125 MHz, $CDCl_3$) 11.28, 18.52, 18.58, 36.16, 46.24, 56.28, 82.54, 111.18, 111.40, 128.12, 128.28, 128.70, 129.08, 132.89, 133.30.

Next, compound **2** (0.5 g, 0.56 mmol) was dried under high vacuum overnight prior to conduction the reaction and was then dissolved in 10mL of dry DCM under argon. The mixture was cooled to 0°C and DIPEA (0.49 mL, 2.8 mmol) was added and allowed to stir for 10 minutes. N,N-diisopropylamino cyanoethyl phosphonamidic-Cl (0.5 mL, 2.25 mmol) was then added in one portion while stirring and the reaction was allowed to warm to room temperature. The reaction was stirred for an additional period of 2 hours until TLC showed completion (30% ethyl acetate/hexanes, v/v). Once complete, the reaction mixture was concentrated, and the residue was dissolved in a small amount of DCM and purified by column chromatography (gradient 0-100% ethyl acetate/hexanes). The appropriate fractions were combined and concentrated to yield compound **3** as a

white solid (0.49 g, 80%). The ^{1}H NMR, ^{13}C NMR, ^{31}P NMR and MS spectra are displayed in **Figures S17-20**. Note: Following the coupling of compound **3** on solid support, the dibrominated DMTr group is cleaved under the conditions used for all other nucleotides (see Materials and Methods).

HRMS calc. [M+Na] 1114.0885. HRMS found ESI+ [M+Na]+ 1114.0877. ^{1}H-NMR δH (500 MHz, $CDCl_3$) 0.86 (d, 3H, J = 6.9 Hz), 1.00 (d, 4H, J = 7.1 Hz), 1.04 (d, 6H, J = 6.8 Hz), 1.07 (d, 3H, J = 6.8 Hz), 1.12 (d, 3H, J = 6.8 Hz), 1.17 (d, 12H, 6.9 Hz), 1.21 (d, 6H, J = 6.8 Hz), 2.00 (m, 1H), 2.41 (m, 1H), 2.53 (m, 1H), 2.71 (m, 2H), 2.78 (m, 3H), 3.21 (dd, 1H, J = 10.8, 4.7 Hz), 3.46-3.65 (m, 12H), 3.78-3.84 (m, 2H), 3.86-3.88 (m, 12H), 3.89-3.94 (m, 2H), 4.12-4.26 (m, 5H), 4.83-4.92 (m, 2H), 5.56 (ddd, 1H, J = 53.6, 5.8, 2.6 Hz), 5.98 (ddd, 1H, J = 54.0, 6.0, 3.9 Hz), 6.16 (dd, 1H, J = 19.5, 3.9 Hz) 6.23 (dd, 1H, J = 21.3, 2.6 Hz), 6.75-6.79 (m, 4H), 7.23-7.27 (m, 7H), 7.29-7.31 (m, 2H), 7.40-7.44 (m, 4H), 7.60 (d, 1H, J = 2.4 Hz), 7.65 (d, 1H, J = 2.4 Hz), 7.67 (d, 1H, J = 2.3 Hz), 7.68 (d, 1H, J = 2.3 Hz). ^{13}C-NMR δ_C (125 MHz, $CDCl_3$) 18.62, 18.67, 18.74, 18.87, 20.10, 20.15, 22.90, 22.92, 22.98, 23.00, 24.32, 24.39, 24.49, 24.55, 24.60, 24.63, 24.68, 35.60, 35.89, 43.19, 43.29, 43.35, 43.45, 45.30, 45.35, 56.25, 56.27, 58.12, 58.16, 62.74, 64.34, 85.30, 85.42, 111.01, 111.06, 111.14, 111.19, 111.29, 116.90, 117.90, 122.42, 122.45, 124.16, 124.73, 127.35, 127.50, 127.99, 128.13, 128.17, 128.37, 128.73, 129.05, 129.10, 129.29, 133.10, 133.15, 133.40, 133.47, 136.69, 137.03, 137.06, 137.32, 143.51, 143.56, 147.85, 147.95, 149.04, 149.29, 154.12, 154.89, 154.95, 154.99, 155.07, 178.95, 179.33. ^{31}P-NMR δP (202 MHz, $CDCl_3$) 14.18 (s), 149.83 (d), 149.77 (d).

Characterization of Compound**s 2** and **3**.

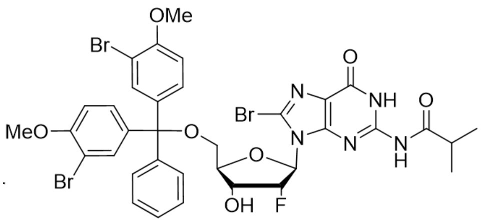

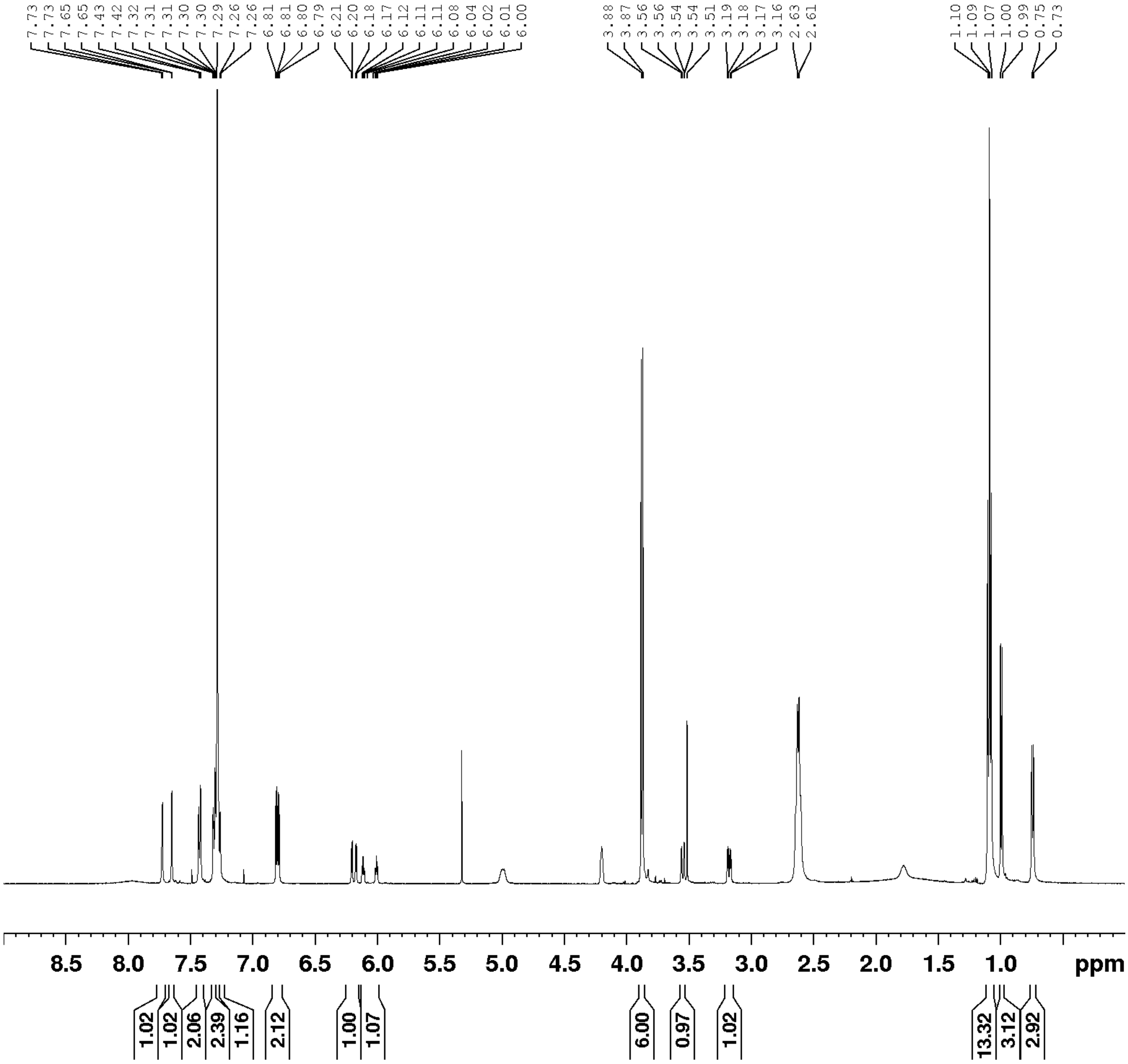

^{1}H NMR spectrum of **compound 2** (500 MHz, $CDCl_3$).

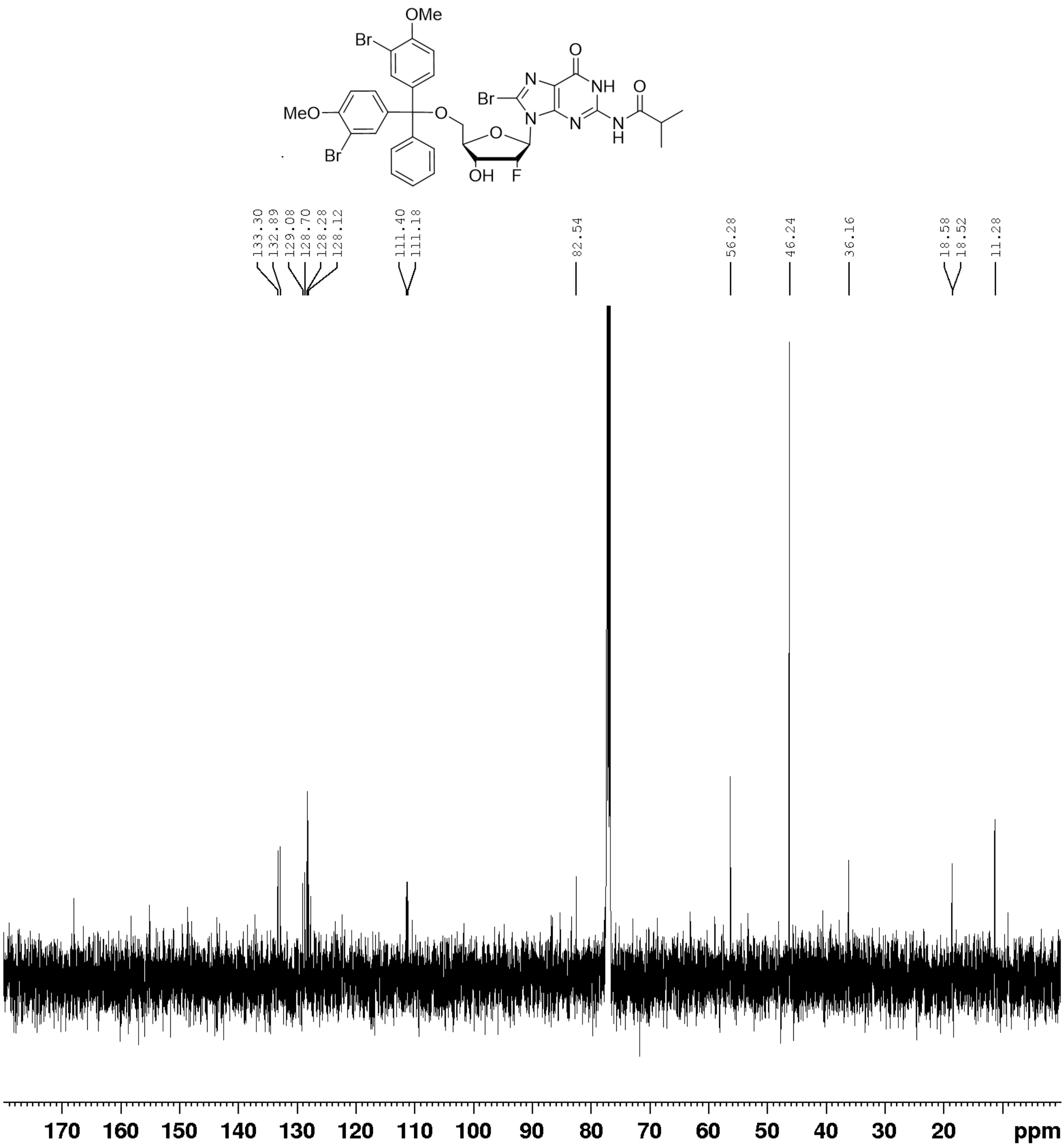

^{13}C NMR spectrum of **compound 2** (125 MHz, $CDCl_3$).

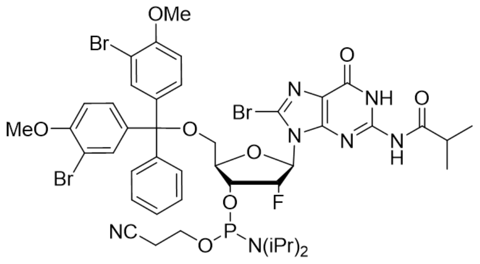

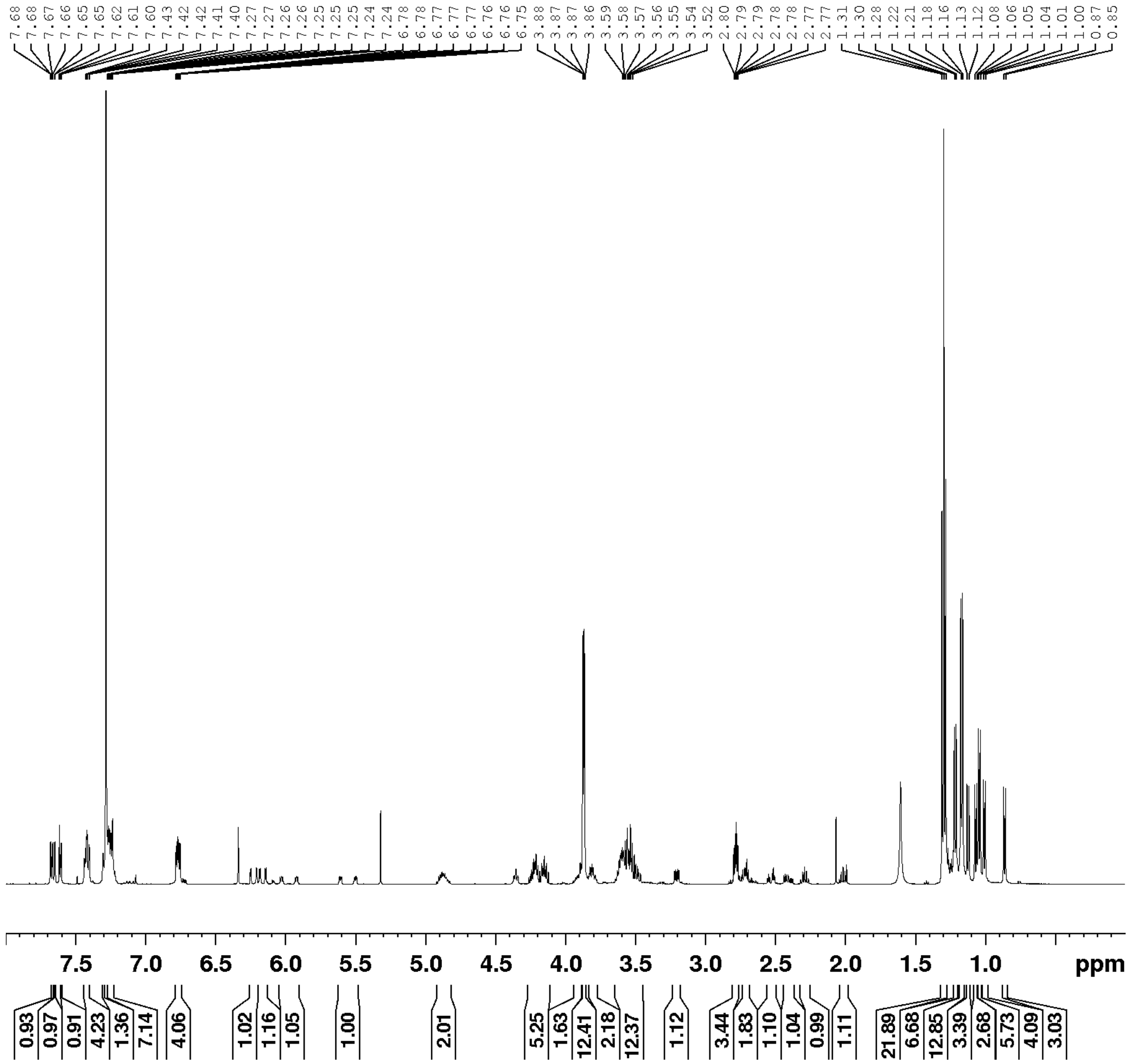

^{1}H NMR spectrum of **compound 3** (500 MHz, $CDCl_3$).

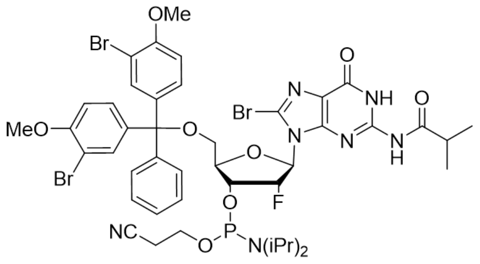

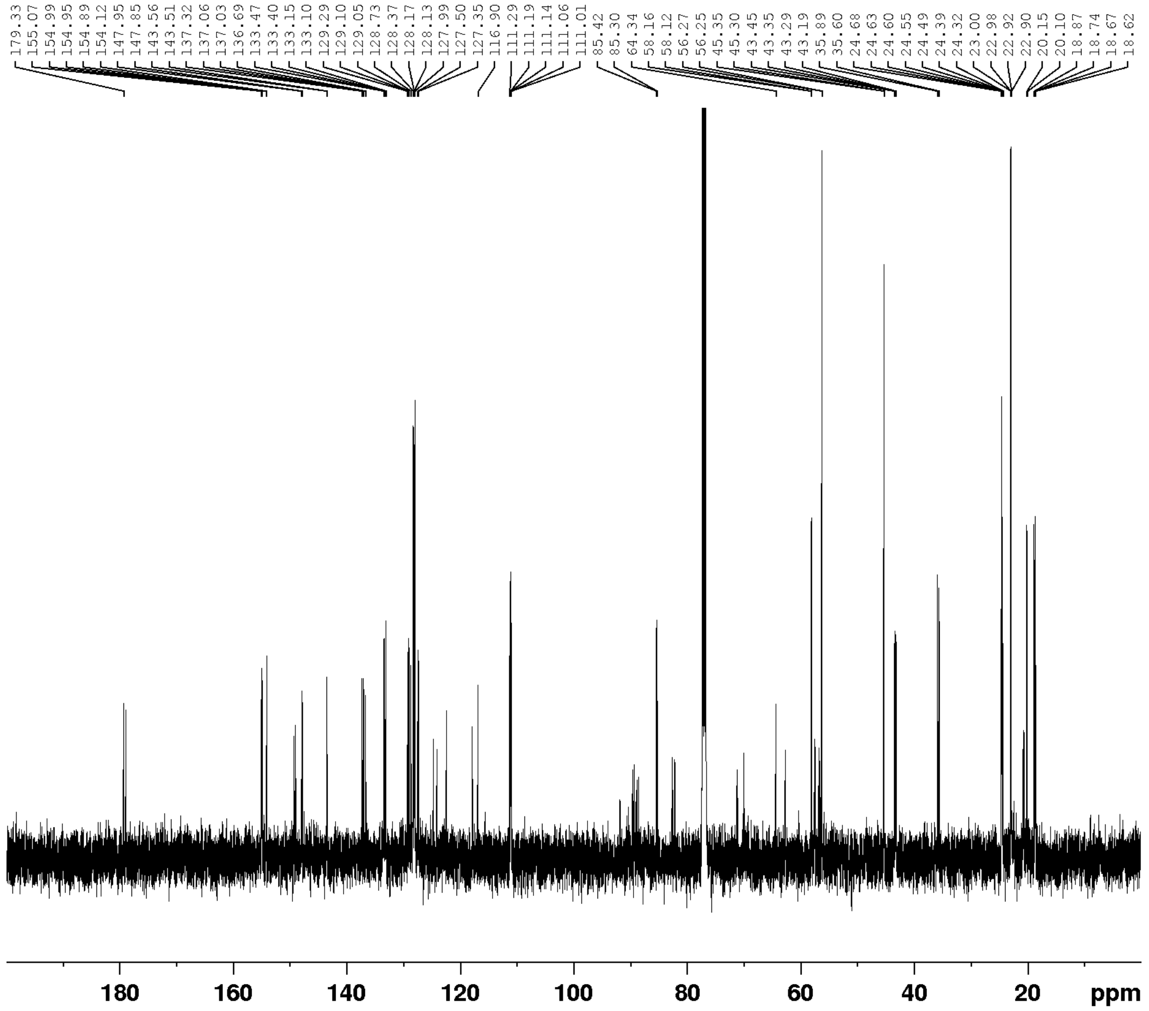

^{13}C NMR spectrum of **compound 3** (125 MHz, $CDCl_3$).

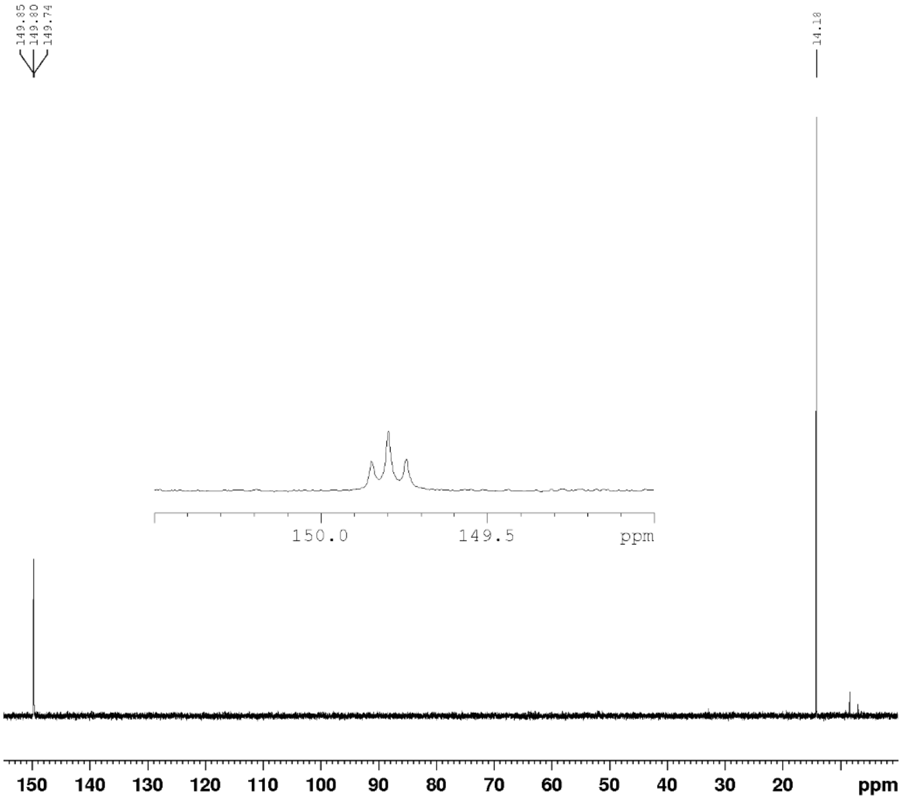

^{31}P NMR spectrum of compound **3** (202 MHz, $CDCl_3$). The apparent triplet observed arises from the overlap of two doublet of doublets. The splitting of ^{31}P-NMR signals arises from long range ^{19}F-^{31}P J coupling as previously observed for other 2'F-nucleoside 3'-O-phosphoramidites.

See, e.g.:
https://doi.org/10.1021/jacs.7b07582
https://doi.org/10.1093%2Fnar%2Fgkl1153
https://doi.org/10.1093/nar/28.18.3625

The signal at ca. 14.2 ppm correspond to the hydrolyzed phosphitylating reagent (H-phosphonate: iPr_2N-PO(H)OCNEt) used in the reaction to prepare compound **3** from compound **2**. This impurity does not interfere during solid-phase synthesis of oligonucleotides.

Mass Spectrum SmartFormula Report

Analysis Info

Analysis Name	D:\Data\Damha\2022-05-17 Damha-Thorpe JAT-BrG-6 ESI +ve.d	Acquisition Date	5/17/2022 3:53:27 PM
Method	Tune_pos_Mid_AW.m	Operator	Alex
Sample Name	2022-05-17 Damha-Thorpe JAT-BrG-6 ESI +ve	Instrument	maXis impact 282001.00044
Comment			

Acquisition Parameter

Source Type	ESI	Ion Polarity	Positive	Set Nebulizer	0.4 Bar
Focus	Not active	Set Capillary	4500 V	Set Dry Heater	180 °C
Scan Begin	200 m/z	Set End Plate Offset	-500 V	Set Dry Gas	4.0 l/min
Scan End	3500 m/z	Set Charging Voltage	2000 V	Set Divert Valve	Source
		Set Corona	0 nA	Set APCI Heater	0 °C

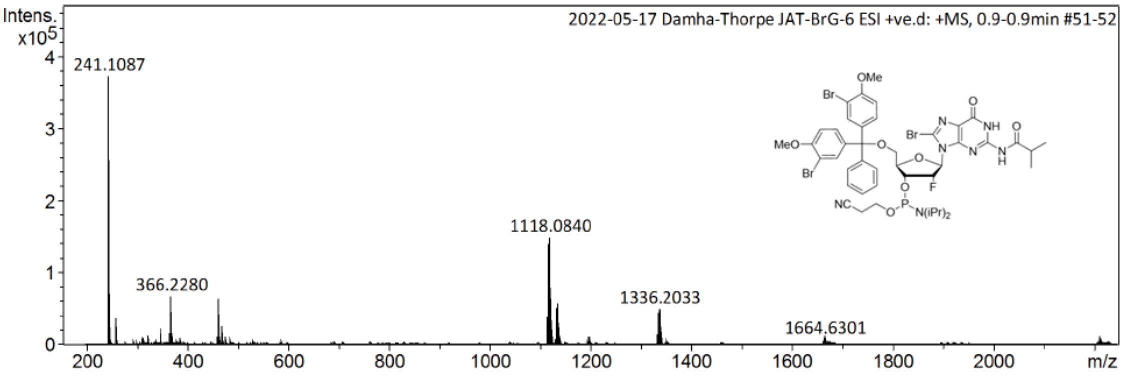

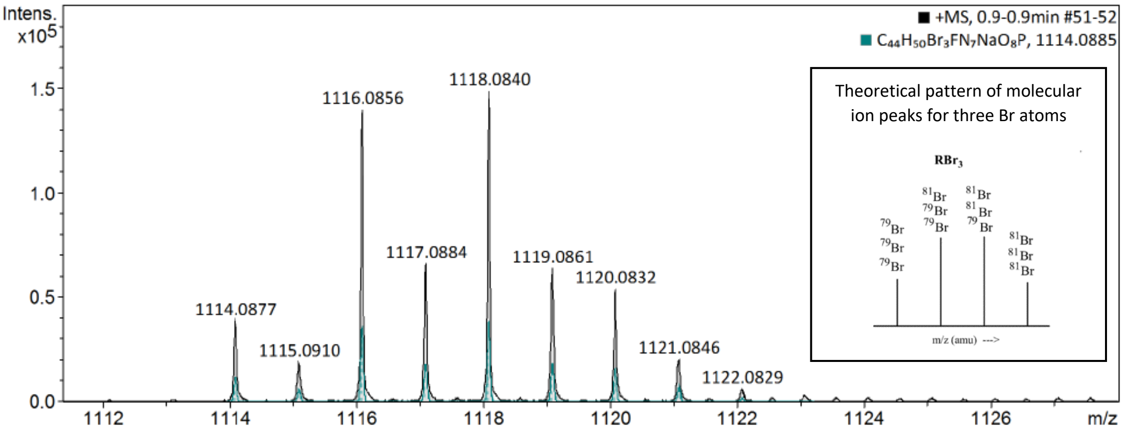

Meas. m/z	#	Ion Formula	m/z	err [ppm]	mSigma	# mSigma	Score	rdb	e⁻ Conf	N-Rule
1114.0877	1	C41H49Br3FN9O10P	1114.0869	-0.7	26.8	1	96.85	20.5	even	ok
	2	C39H37Br3FN23P	1114.0869	-0.7	30.9	2	87.31	31.5	even	ok
	3	C42H45Br3FN13O6P	1114.0882	0.5	31.1	3	100.00	25.5	even	ok
	4	C57H49Br3FNO5P	1114.0877	0.0	69.5	4	38.58	32.5	even	ok
	1	C43H54Br3FN3NaO12P	1114.0871	-0.5	27.3	1	100.00	16.5	even	ok
	2	C41H42Br3FN17NaO2P	1114.0871	-0.5	31.2	2	90.54	27.5	even	ok
	3	C44H50Br3FN7NaO8P	1114.0885	0.7	31.6	3	77.48	21.5	even	ok
	4	C56H46Br3FN5NaOP	1114.0866	-0.9	69.4	4	21.45	34.5	even	ok
	1	C38H46Br3FKN17O3P	1114.0873	-0.3	35.0	1	93.11	22.5	even	ok
	2	C41H54Br3FKN7O9P	1114.0886	0.9	36.4	2	73.77	16.5	even	ok
	3	C40H58Br3FKN3O13P	1114.0873	-0.3	36.5	3	100.00	11.5	even	ok
	4	C53H50Br3FKN5O2P	1114.0868	-0.8	64.7	4	32.45	29.5	even	ok

ESI-Mass spectrum of compound **3**. A compound with three $^{79}Br/^{81}Br$ atoms will have four major isotope peaks, overlapping at every two m/z units (see inset). The green trace is the theoretical isotope pattern for the formula derived from the data. Furthermore, the mSigma values indicate how close the measured isotope pattern matches the theoretical pattern. mSigma values less than 50 are acceptable. For a compound of 44 carbons (MW >1000) the contribution from ^{13}C adds more isotope peaks. This data together with the exact mass and isotope pattern provide confident confirmation for this ion.
